# Broad-range capsule-dependent lytic *Sugarlandvirus* against *Klebsiella* sp.

**DOI:** 10.1128/spectrum.04298-22

**Published:** 2023-10-26

**Authors:** Robby Concha-Eloko, Pilar Barberán-Martínez, Rafael Sanjuán, Pilar Domingo-Calap

**Affiliations:** 1 Instituto de Biología Integrativa de Sistemas, Universitat de València-CSIC, Paterna, Spain; Memorial Sloan Kettering Cancer Center, New York, USA

**Keywords:** *Klebsiella*, bacteriophages, capsule, phage therapy, infection range, phage cocktail

## Abstract

The emergence of antibiotic-resistant bacteria has become a serious global health threat requiring the development of novel treatments. *Klebsiella pneumoniae* is an encapsulated bacterium considered a major concern due to its high resistance, prevalence, and mortality rates. Phage therapy has been proposed as a very promising alternative to combat infections by *Klebsiella* sp. infections. However, most of the *Klebsiella* phages described so far present a high specificity, infecting one or a few capsular types due to the presence of depolymerases in their genomes, which limits their therapeutic potential. Here, we present three new *Klebsiella* phages isolated from the environment, vB_Kpn_K7PH164C4, vB_Kpn_K30λ2.2, and vB_Kpl_K32PH164C1, belonging to the family *Demerecviridae* and the genus *Sugarlandvirus*. The most important feature of these new *Klebsiella* phages is their broad host range, especially vB_Kpn_K7PH164C4 and vB_Kpn_K30λ2.2, which infects strains of more than 20 different capsular types, representing the broadest infection range observed for *Klebsiella* phages. Genomic analysis revealed the presence of three receptor-binding proteins lacking depolymerase domains. Nevertheless, capsule expression is suggested to be a determining factor in phage infectivity, despite the absence of depolymerase activity against capsular components. Our findings hold potential for the development of promising phage-based therapeutics directed against *K. pneumoniae*.

**IMPORTANCE:**

The emergence of multi-drug resistant bacteria is a global health problem. Among them, *Klebsiella pneumoniae* is considered a high-priority pathogen, making it necessary to develop new therapeutic tools to reduce the bacterial burden in an effective and sustainable manner. Phages, bacterial viruses, are very promising tools. However, phages are highy specific, rendering large-scale therapeutics costly to implement. This is especially certain in *Klebsiella*, a capsular bacterium in which phages have been shown to be capsular type dependent, infecting one or a few capsular types through specific enzymes called depolymerases. In this study, we have isolated and characterized novel phages with lytic ability against bacteria from a wide variety of capsular types, representing the *Klebsiella* phages with the widest range of infection described. Remarkably, these broad-range phages showed capsule dependency, despite the absence of depolymerases in their genomes, implying that infectivity could be governed by alternative mechanisms yet to be uncovered.

## INTRODUCTION

Anti-microbial resistance is one of the most important problems for global public health (1). Indeed, the emergence of multi-drug-resistant (MDR) bacteria is a major concern due to the high morbidity and mortality rates associated with them. Recently, it has been shown that in 2019, more than 1.2 million deaths were attributable to MDR bacteria worldwide (2). Among them, *Klebsiella pneumoniae* was one of the pathogens leading resistance-associated deaths. Although *K. pneumoniae* is a commensal bacterium in healthy individuals, it may be implicated in nosocomial infections, especially in immunosuppressed patients. It can cause a wide range of severe infections in the urinary tract, lungs, liver, or brain (3). Several virulence factors have been described, the production of a capsular polysaccharide (CPS) being the most relevant (4). Indeed, CPS can act as a barrier for the bacterium against certain molecules, such as proteins and antibiotics, or against the immune system (5). In fact, *Klebsiella* sp. is a highly variable genus, mainly due to modifications of CPS. Briefly, 77 serotypes were initially identified, which are considered as the reference capsular types (K-types) (6). However, thanks to genotyping, new K loci have been discovered and, as a result, there are more than 100 K-types (7). Interestingly, different *Klebsiella* spp. may share K-types, indicating gene transfer between bacteria (8).

The emergence of MDR *Klebsiella* strains worldwide is a major challenge, particularly carbapenem-resistant ones, which urgently requires alternative anti-microbials. In this scenario, bacteriophage (phage) therapy is considered a very promising alternative (9). Phages, bacterial viruses, are highly specific. They recognize bacteria by attaching to the host through the receptor-binding protein (RBP), which recognizes a receptor on the host surface, followed by injection of the genetic material into the host. In lytic phages, after evading host defenses, the phage exploits the host machinery to replicate and produce progeny after bursting the host cell. In contrast, lysogenic phages can integrate their genome into the bacterial one and replicate without producing viral progeny. Phage therapy focuses mainly on lytic phages since they lyse bacteria more efficiently and are less likely to promote horizontal transfer of antibiotic resistance genes or bacterial virulence factors (10).

Most studies emphasize phage attachment to the host as a major determinant of host tropism (11). The ability of the phage to infect diverse hosts, known as host range, is influenced by the sequence of the RBP (12). Indeed, phage evolution can result in changes in the RBP sequence that can alter or enhance the ability of a given phage to infect a host (13 – 15). Thus, a major limitation to the success of phage therapy is the emergence of host resistance to the phage through modification of the phage receptor (16). Both narrow- and broad-range phages present interesting features for phage therapy. Narrow-range phages have the ability to target very specific bacteria at the species or the strain level, leading to personalized medicine. In contrast, broad-range phages can infect multiple strains or species, being particularly useful when little information about the pathogen can be gathered (9, 16). In *Klebsiella* sp., the enormous diversity of K-types has been associated with more specialized phages encoding specific enzymes called depolymerases that can degrade targeted exopolysaccharides present in the bacterial capsule. Indeed, such depolymerase domains are usually found in the phage RBP (17), and some can recognize multiple *Klebsiella* strains or species (17 – 19).

Until recently, broad-range *Klebsiella* phages were not common and were associated with multiple depolymerase domains present in the phage genome, as observed for ØK64-1 *Klebsiella* phage, which contains nine functional genes encoding capsular depolymerases. Recently, however, new broad-range phages without depolymerase domains have been described, which are capable of infecting about a dozen different K-types (20), suggesting a different mechanism of infection. The discovery of new broad-spectrum phages is of great importance for the development and implementation of new control strategies. In addition, it allows increase of the knowledge of the diversity of phages in nature. However, isolating broad-spectrum phages is not an easy task, especially because of the specificity of *Klebsiella* phages by capsular type. Therefore, bioprospecting is important to isolate new broad-spectrum phages and to characterize them at the molecular level to understand the mechanisms of infection, with emphasis on the capsule. Here we present three novel *Klebsiella* phages isolated in *K. pneumoniae* and *Klebsiella planticola* strains. We will focus on two of them, vB_Kpn_K7PH164C4 and vB_Kpn_K30λ2.2, which present the broadest host range reported to date. These phages presented neither a genetic association with a lysogenic lifestyle nor a depolymerase sequence in their genome, thus presenting interesting features for development of phage-based anti-*Klebsiella* therapeutics.

## RESULTS

### Isolation and phenotypic characterization of three new *Klebsiella* phages

**Fig 1:**
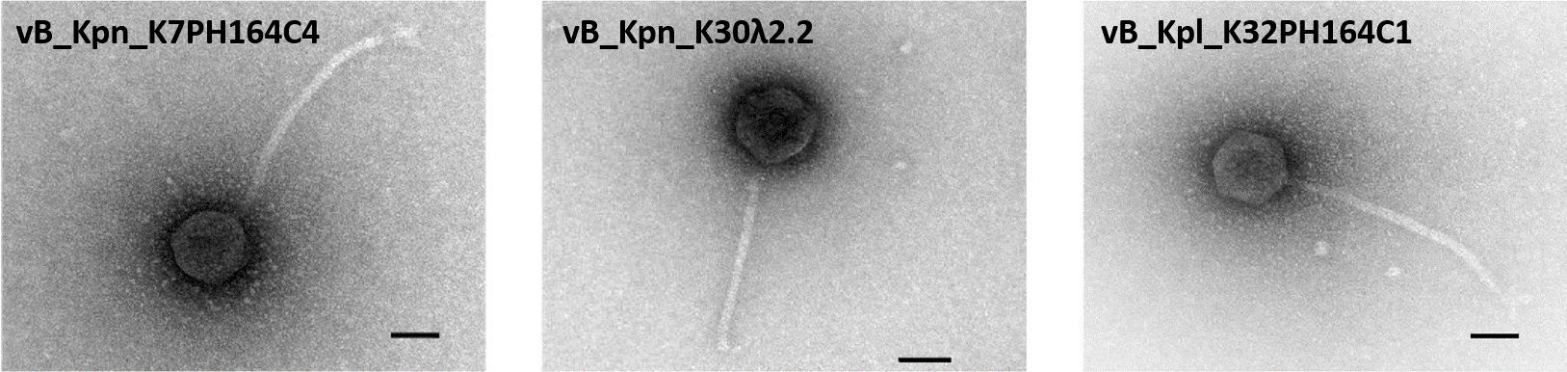
Transmission electron micrographs of *Klebsiella* phages vB_Kpn_K7PH164C4, vB_Kpn_K30λ2.2, and vB_Kpl_K32PH164C1. Scale bar: 50 nm.

Filtered sewage samples from the metropolitan area of Valencia (Spain) were tested on semi-solidified soft agar using different reference host *Klebsiella* strains from the collection of the Statens Serum Institute (Copenhagen, Denmark). Three new *Klebsiella* phages, vB_Kpn_K7PH164C4, vB_Kpn_K30λ2.2, and vB_Kpl_K32PH164C1, were isolated and characterized. Phage vB_Kpn_K7PH164C4 was isolated in *K. pneumoniae* Aerogenes 4140 (K-type 7, a pathogenic capsular type). Phage vB_Kpn_K30λ2.2 was isolated in *K. pneumoniae* 7824 (K-type 30, one of the most prevalent capsular types). Finally, phage vB_Kpl_K32PH164C1 was isolated in *K. planticola* 6837 (K-type 32). Phages were purified by three plaque-to-plaque passages and amplified in liquid media, reaching a titer of 4.5 × 10^9^ plaque-forming unit (PFU)/mL for vB_Kpn_K7PH164C4, 1.5 × 10^10^ PFU/mL for K30λ2.2, and 1.6 × 10^9^ PFU/mL for vB_Kpl_K32PH164C1. Despite being isolated in different hosts (including different *Klebsiella* species) and from different sewage samples, all three phages had similar plaque morphology in terms of small size, turbidity, and absence of halo. High-titer phages were obtained through high-speed centrifugation and observed by transmission electron microscopy. Micrographs showed a similar morphology for the three isolated phages, with an icosahedral capsid and a long tail, ranging from 200 and 250 nm (Fig. 1).

### Determination of the host range of the new *Klebsiella* phages

**Fig 2:**
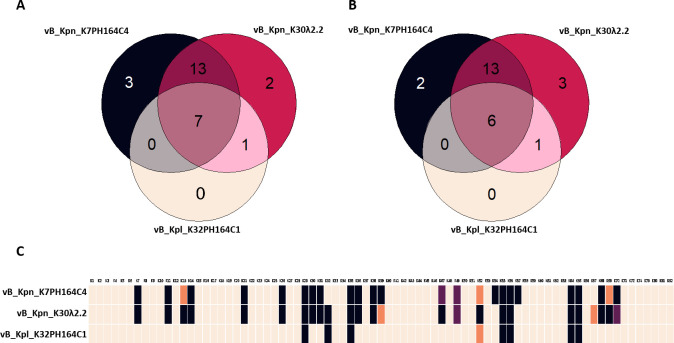
Host range comparison of *Klebsiella* phages vB_Kpn_K7PH164C4, vB_Kpn_K30λ2.2, and vB_Kpl_K32PH164C1. Venn diagram of susceptible K-types in (**A) **solid media or (**B**) liquid culture for the three isolated phages. (**C**) Infectivity matrix for *Klebsiella* phages vB_Kpn_K7PH164C4 and vB_Kpn_K30λ2.2 in the 77 reference *Klebsiella* capsular types. Black denotes positive infection in liquid media and semi-solid media. Purple denotes positive infection in liquid media. Orange indicates positive infection in semi-solid media. Light orange indicates no infection.

To assess the host range of the three new *Klebsiella* phages, infectivity assays were performed on the 77 reference K-types available in the *Klebsiella* species collection of the Statens Serum Institute. The assays were performed by spot tests on semi-solid cultures, obtaining an infectivity matrix for each phage. All three phages were capable of infecting multiple K-types, indicating their broad-spectrum nature (Fig. 2A). *Klebsiella* phage vB_Kpl_K32PH164C1 infected strains of eight different capsular types. The *Klebsiella* phages vB_Kpn_K7PH164C4 and vB_Kpn_K30λ2.2 displayed higher infectivity, infecting strains of 23 different capsular types in semi-solid media. The infection profiles of the three phages were largely coincident. The *Klebsiella* phage vB_Kpl_K32PH164C1 was able to infect strains of K-types K29, K32, K35, K52, K55, K56, K64, and K65. Among these strains, all were also susceptible to vB_Kpn_K30λ2.2, and seven (all except the K-type K32 strain) were susceptible to vB_Kpn_K7PH164C4. In addition, other strains of 13 K-types (K7, K11, K13, K14, K21, K26, K30, K31, K36, K38, K39, K68, and K69) were susceptible to both vB_Kpn_K7PH164C4 and vB_Kpn_K30λ2.2. Finally, among the isolated phages, some strains were susceptible exclusively to one of them. Strains of K-types K54, K57, and K70 were susceptible exclusively to vB_Kpn_K7PH164C4, whereas strains of K-types K47 and K67 were susceptible only to vB_Kpn_K30λ2.2.

### Infectivity and lytic activity of broad-range *Klebsiella* phages in liquid cultures

The *Klebsiella* phages vB_Kpn_K30λ2.2 and vB_Kpn_K7PH164C4 were found to be broad-spectrum phages in semi-solid media. To determine their infectivity and killing efficacy in liquid cultures, vB_Kpn_K30λ2.2 and vB_Kpn_K7PH164C4 were tested against the 77 reference K-types in liquid cultures (Fig. 2B; Table S1). Most of the results obtained by spot tests in semi-solid media matched with those obtained in liquid cultures. However, a few differences between the two methods were observed (Fig. 2C). The *Klebsiella* phage vB_Kpn_K7PH164C4 was able to infect strains of 23 K-types in semi-solid media versus 22 K-types in liquid culture. Differences were found for strains of K-types K13, K52, and K69, as infectivity was observed in petri dishes but not detected in liquid cultures. In the case of strains of K-types K47 and K49, a reduction of optical density (OD) in liquid was observed in the presence of the phage, while no lysis occurred in spots. In addition, *Klebsiella* phage vB_Kpn_K30λ2.2 was able to infect strains of 23 K-types in liquid. In this case, the exceptions concerned strains of K-types K39 and K67, in which no differences were detected in the liquid between infected and uninfected cultures and between strains of K-types K49 and K70, in which no lysis was observed by the spot test.

**Fig 3:**
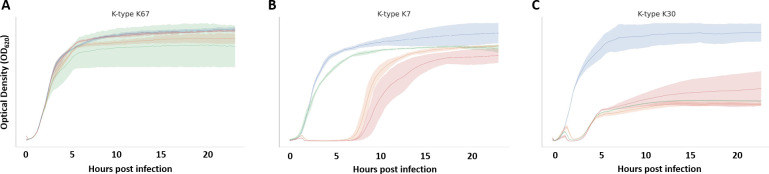
Representative lysis assays in liquid cultures of *Klebsiella* phages. (**A**) Phages are not infective in the tested bacteria. (**B**) Phages can infect the bacterial strain, but the emergence of resistant bacteria occurs within a few hours. (**C**) Phages infect the tested bacteria, but the emergence of resistance is delayed or reduced. Blue indicates bacteria. Orange denotes bacteria + phage vB_Kpn_K7PH164C4. Green denotes bacteria + phage vB_Kpn_K30λ2.2. Red denotes bacteria + phage cocktail vB_Kpn_K7PH164C4 and vB_Kpn_K30λ2.2. All assays were performed in triplicate.

At least three main types of kinetics were distinguished among the cultures tested. In most cases, no infectivity was assumed when the curves between the bacterial strains in the presence and in the absence of the phages were similar (Fig. 3A). The second type corresponded to kinetics that initiate bacterial growth but quickly decrease OD due to phage lysis, followed by a phase of emergence of phage-resistant bacteria in the cultures, reaching similar values of OD to the non-inoculated culture. The emergence of phage-resistant bacterial clones was observed after a short period of time in both phages, ranging between 2 and 8 h, with the strain from the K-type K7 being the one in which phage-resistant bacteria emerged earlier (Fig. 3B). Finally, a third type of kinetics was observed when the cultures inoculated with the phages were not able to recover the OD of the non-inoculated cultures, reaching a plateau at lower OD (Fig. 3C).

Finally, the bactericidal activity of the phages was tested against strains for which a decrease in OD was reported. Bacterial death was confirmed in the presence and absence of the phages after 4 and 18 h postinfection (hpi), and measured as colony forming units (CFU) counts. The results confirmed, in most cases, the observed reduction in OD of the cultures (Table S2), supporting the bactericidal activity of the phages against a wide range of *Klebsiella* capsular types.

### Phage cocktail encompassing broad-range *Klebsiella* phages

The combination of the two broad-range *Klebsiella* phages vB_Kpn_K30λ2.2 and vB_Kpn_K7PH164C4 was tested in liquid infections in order to evaluate phage-phage interactions for future implementation of phage cocktails (Fig. S1). The cocktail was tested on K-types that were susceptible to at least one phage in liquid or semi-solid media. Attending to the OD curves, for most K-types, we found that the phage cocktail was not significantly different from the most efficient phage taken alone (*t*-test *P* value > 0.5). However, for K-types K13 and K47, the cocktail was significantly more performant than phage alone based on infection kinetics. The area under the curve (AUC) for the cocktail was significantly lower in K13 than the AUC for vB_Kpn_K7PH164C4 (*t*-test *P* value = 0.008) and for vB_Kpn_K30λ2.2 (*t*-test *P* value = 0.013). In addition, the AUC for the cocktail was significantly lower in K47 than the AUC for vB_Kpn_K7PH164C4 (*t*-test *P* value = 0.044) and for vB_Kpn_K30λ2.2 (*t*-test *P* value = 0.021). Thus, the combination of the phages resulted in a slight improvement in K13 and K47. To evaluate the bactericidal activity of the phage cocktail, CFU counts were performed at 4 and 18 hpi. However, the results obtained were not significant in terms of viable bacteria after infection (Table S2).

### Genomic analysis of the new *Klebsiella *phages

**Fig 4:**
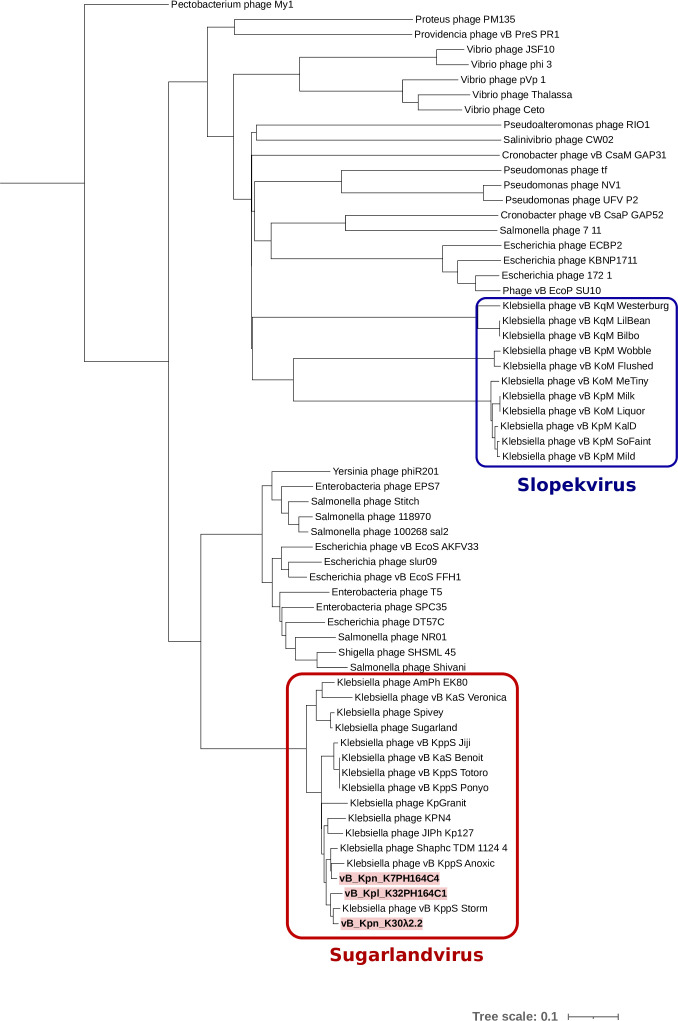
Phylogenetic tree of phages vB_Kpn_K7PH164C4, vB_Kpn_K30λ2.2, and vB_Kpl_K32PH164C1 with closely related sequences and other phage genera isolated in *Klebsiella*. Phage labels in red correspond to the phages described in this study. Numbers indicate bootstrap values (100 pseudo-bootstrap replicates).

High-titer lysates were used to extract phage DNAs and sequenced using MiSeq Illumina platform. The *Klebsiella* phages vB_Kpn_K7PH164C4, vB_Kpn_K30λ2.2, and vB_Kpl_K32PH164C1 were assembled into 113,206-, 112,044- and 110,740-bp contigs, respectively (ENA PRJEB53659, Table 1). Genomic similarity among the three phages ranged from 89.4% to 91.4%, revealing high homology. Taxonomic predictions assigned the isolated phages to the family *Demerecviridae* and the genus *Sugarlandvirus*. Comparison of the genomes of the isolated phages with those of previously described sugarlandviruses showed that the phage vB_Kpn_K30λ2.2 and *vBKppS Storm* belong to the same species with 95.6% similarity, while the other two phages represent new species. The GC content ranged from 44.8% to 45.5%, similar to that of most sugarlandviruses. The relationship between the newly isolated phages and the genus *Sugarlandvirus* was also confirmed by phylogenetic analysis (Fig. 4; Fig. S2). Indeed, the isolated phages, together with the previously described sugarlandviruses, were exclusively grouped within a distinct taxonomic clade.

**TABLE 1:** Genomic characteristics of the *Klebsiella* phages vB_Kpn_K7PH164C4, vB_Kpn_K30λ2.2, and vB_Kpl_K32PH164C1

Klebsiella phage	Genome length (in base pair)	Average sequencing coverage	GC content (%)	DNA sequences		Average nucleotide identity (%)			
				CDS (hypothetical)	tRNA	vB_Kpn_K7PH164C4	vB_Kpn_K30λ2.2	vB_Kpl_K32PH164C1	vBKppS storm
*vB_Kpn_K7PH164C4*	113,206	118.23	45.476	152 (94)	27	–^*a*^			
*vB_Kpn_K30λ2.2*	112,044	118.33	45.446	152 (96)	26	90.249	–		
*vB_Kpl_K32PH164C1*	110,740	26.91	45.367	151 (93)	27	89.398	91.377	–	
*vBKppS Storm*	110,834	–	45.557	151 (93)	26	90.083	95.625	91.332	–
*AmPh EK80*	112,215	–	44.816	163	27	81.446	81.224	81.242	80.364
*JIPh Kp127*	113,671	–	45.267	154	26	88.407	87.116	87.305	86.032
*KpGranit*	122,710	–	45.216	166	28	90.703	90.64	89.642	88.49
*KPN4*	108,916	–	45.584	148	28	86.946	87.438	87.587	88.318
*Shaphc TDM 1124–4*	112,841	–	45.538	152	27	93.439	91.041	89.194	90.829
*Spivey*	110,659	–	44.911	162	28	82.927	83.423	83.191	81.738
*Sugarland*	111,103	–	44.958	160	28	83.793	84.232	83.128	82.404
*vBKaSBenoit*	109,017	–	45.369	153	26	87.613	88.367	87.266	87.102
*vBKaS Veronica*	110,196	–	44.851	158	23	78.178	76.996	76.92	75.65
*vBKppSAnoxic*	109,500	–	45.478	156	24	92.332	89.145	88.58	89.207
*vBKppSJiji*	113,155	–	45.472	151	26	88.602	89.302	88.186	88.083
*vBKppSPonyo*	109,014	–	45.368	153	26	87.614	88.369	87.268	87.103
*vBKppSTotoro*	109,014	–	45.368	153	26	87.613	88.368	87.267	87.102

^*a*^ –, Not available.

**Fig 5:**
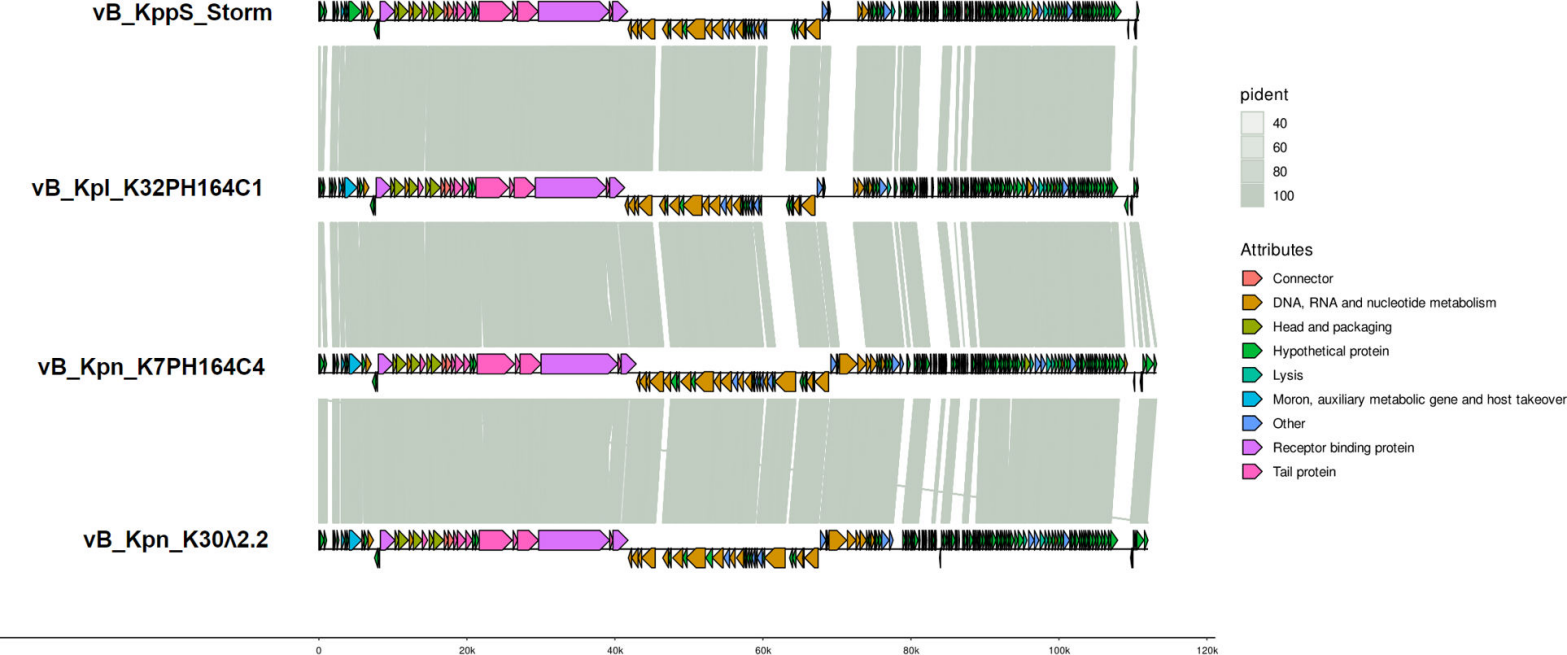
Comparative genomics of isolated phages vB_Kpn_K7PH164C4, vB_Kpn_K30λ2.2, and vB_Kpl_K32PH164C1, and a previously described *Sugarlandvirus* vB KppS Storm. Genes within consecutive genomes were compared using blastp.

Genomic annotation yielded between 151 and 152 predicted open reading frames (ORFs) with the multiPhATE2 pipeline, and between 26 and 27 predicted tRNAs (Fig. 5). About 62% of the ORFs were hypothetical proteins or of unknown function (94/152 for vB_Kpn_K7PH164C4, 96/152 for vB_Kpn_K30λ2.2, and 93/151 for vB_Kpl_K32PH164C1). Among the others, some were predicted to be involved in phage structure (major capsid protein, small and large terminase subunits) and, in particular, in the tail (tail tip protein, major and minor tail proteins, baseplate protein, and phage tail length tape-measure protein). In addition, DNA, RNA, and nucleotide metabolism (DNA polymerase, DNA primase C, DNA primase-helicase, and subunits A and B of DNA ligase), lysis (I spanin, endolysin, holin, and cell wall hydrolase), host recognition (tail fiber proteins), metabolic genes, and host takeover (A1 and A2 proteins) were found. No depolymerase sequences could be identified by the methods used. The complete list of the predicted ORFs, as well as their transcription orientation, can be found in Table S3. The life cycle of phages vB_Kpn_K7PH164C4, vB_Kpn_K30λ2.2, and vB_Kpl_K32PH164C1 was predicted to be virulent with 99.73%, 99.65%, and 100%, respectively. Additionally, no genes associated with a temperate life cycle, such as a recombinase, integrase, excisionase, or transposase, were identified in the isolated genomes.

The sequences encoding endolysin and holin were very similar in all the three phages, showing 99% identity. However, some differences were identified in the genes encoding RBP. The three isolated phages harbored three RBP coding sequences, which we will refer to as RBP α, RBP β, and RBP γ (Fig. S3). In particular, RBP α and RBP γ exhibited very high levels of identity ranging from 97.42% to 98.48% and from 93.61% to 97.03%, respectively, among the three isolated phages (Table 2). RBP β was the most divergent, as vB_Kpn_K30λ2.2 together with vB_Kpn_K7PH164C4 and vB_Kpl_K32PH164C1 together with vB_Kpn_K7PH164C4, respectively, shared 78.9% and 79.21%, while vB_Kpl_K32PH164C1 and vB_Kpn_K30λ2.2 presented 98.32%. Interestingly, no depolymerase-related genes were found in any phage, which correlated with the absence of halos on the plaques. To further characterize the functionalities of the identified RBPs, domain annotation was performed using Interproscan (version 92.0) (https://www.ebi.ac.uk/interpro/, accessed in February 2023) (21). Surprisingly, no annotated domain was identified in either RBP α or the RBP γ of the isolated phages. However, RBP β of the three isolated phages harbored domains homologous to the IPR008979 and IPR008160 entries, corresponding to the “galactose-binding-like” (GBL) domain superfamily, and the “collagen triple helix repeat” domain, respectively. The GBL domain was conserved in all three sequences, with an initial position between 1164 and 1167 and an ending position between 1312 and 1315. A first collagen triple helix repeat domain was present in all the three phages sequences, with an initial position between 1911 and 1917 and an ending position between 1960 and 1968. Interestingly, a second collagen triple helix repeat domain was identified in the RBP β coding sequence in the phages vB_Kpn_K30λ2.2 and vB_Kpl_K32PH164C1 with a starting position ranging from 3019 to 3022 and an ending position ranging from 3077 to 3080 (Table S4). Finally, three-dimensional (3D) structure prediction of phage RBPs was performed not only to provide amino acid sequence differences, but also to determine differences in the host range. In RBP α and the RBP γ, sites with evidence of positive selection pressure were identified. However, no differences were observed between the isolated phages at the corresponding positions (data not shown).

**TABLE 2:** Receptor-binding protein (RBP) comparison between the *Klebsiella* phages vB_Kpn_K7PH164C4, vB_Kpn_K30λ2.2, and vB_Kpl_K32PH164C1 and the related phage vB_KppS_Storm^*a*^

RBP percent identity (α/β/γ)				
	*vB_Kpl_K32PH164C1*	*vB_Kpn_K30λ2.2*	*vB_Kpn_K7PH164C4*	*vBKppS Storm*
*vB_Kpl_K32PH164C1*	–			
*vB_Kpn_K30λ2.2*	98.33/97.03/98.38	–		
*vB_Kpn_K7PH164C4*	97.42/93.61/79.21	98.48/94.03/78.92	–	
*vBKppS Storm*	98.18/96.96/98.09	99.54/99.88/99.71	98.33/94.00/78.77	–

^*a*^ RBP percent identity is shown for each of RBP α, RBP β, and RBP γ.

### Effect of capsule expression on phage susceptibility

**Fig 6:**
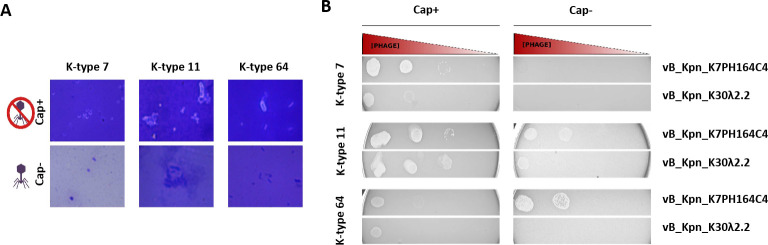
Determination of effect of *Klebsiella* capsule expression on phage susceptibility. (**A**) Microscope photographs after capsule staining in the wild-type and cap− bacteria in K7, K11, and K64. (**B**) Spot test results obtained for vB_Kpn_K7PH164C4 and vB_Kpn_K30λ2.2 in cap− and wild-type bacteria in K7, K11, and K64.

To determine whether the capsule was involved in the infection process, three reference capsular (cap+) strains (cap+ K7, cap+ K11, and cap+ K64) were randomly chosen to generate acapsular (cap−) bacteria. The susceptibility of the cap− bacteria to the two broad-range *Klebsiella* phages was assessed through serial dilutions on spot test (Fig. 6). Spot tests on cap+ K 7 revealed lytic plaques in vB_Kpn_K7PH164C4 and vB_Kpn_K30λ2.2. However, no plaques were observed on the cap− K7. Spot tests on the cap+ K64 revealed lytic plaques for both phages, but phages showed reduced efficiency of plating (EOP) in cap− K64. Finally, spot tests on cap+ K11 showed lytic plaques in both phages, but surprisingly, phage vB_Kpn_K7PH164C4 showed higher EOP against the cap− K11, while no infection was observed for vB_Kpn_K30λ2.2. These results suggested that either the phages were unable to infect cap− bacteria or their infectivity was strongly reduced, suggesting capsule dependence. However, in K11, phage vB_Kpn_K7PH164C4 was able to infect both cap+ and cap− bacteria, suggesting alternative mechanisms of infection.

### Association between the bacterial genetic sequence at the K-locus and susceptibility to phages

Phages primarily target oligosaccharides and lipopolysaccharide (LPS) repeats found in the CPS of bacteria. To elucidate the interactions between the isolated phages and the CPS, a gene presence matrix was generated for the K-locus. Genes were evaluated based on two criteria: evidence of their influence on capsule expression and presence in at least five strains. The association between phage susceptibility and the subset of genes involved in CPS regulation was then examined. For both vB_Kpn_K7PH164C4 and vB_Kpn_K30λ2.2, a gene encoding the PAP2 phosphatase family protein was significantly associated with bacterial infection (Fisher test, *P* value of < 0.001, and Bonferroni correction, *P* value = 0.008, after false discovery rate correction). All bacteria carrying this gene were susceptible to both *Sugarlandvirus* phages (6 of 6) (Fig. S4A). In addition, a gene encoding the outer membrane protein Wzi showed an association with bacterial infection for both phages (*P* value = 0.037 for the *t*-test). Notably, this gene was found in three strains susceptible to both phages, but none of them carried the PAP2 gene (Fig. S4B).

## DISCUSSION

Three new broad-range *Klebsiella* sugarlandviruses, vB_Kpn_K7PH164C4, vB_Kpn_K30λ2.2, and vB_Kpl_K32PH164C1, were isolated from wastewater using different *Klebsiella* strains as target hosts. In fact, vB_Kpn_K7PH164C4 and vB_Kpn_K30λ2.2 covered, to our knowledge, the widest range of K-types in *Klebsiella*, with more than 20 susceptible K-types each. Among other broad-range *Klebsiella* phages, ΦK64-1 could infect up to nine K-types, mainly due to the presence of multiple depolymerases (18). More recently, the *Slopekvirus* vB_KoM-MeTiny, covering a host range of 12 different K-types (20), and two other *Slopekvirus* presenting lytic activity against 66% of bacteria from a panel of 108 strains (22) have been described. Although the *Sugarlandvirus* and *Slopekvirus* genera comprise phages of broad host range isolated in *Klebsiella,* they appear to be relatively phylogenetically distant. Phages used in the phylogenetic analysis that belonged to the two genera formed two groups located in each of the major clades of the tree. Moreover, phages isolated in different genera of bacteria such as *Escherichia*, *Salmonella*, *Yersinia*, *Shigella*, or *Enterobacteria* were more closely related to *Sugarlandvirus* than *Slopekvirus*. This is congruent with the observation that phages could potentially infect hosts from different genera (23, 24). However, these large host range differences between the phages presented here and those previously described above could be explained by the larger panel of bacteria tested. Indeed, the phages vB_Kpn_K7PH164C4 and vB_Kpn_K30λ2.2 were tested on some available isolates of *Salmonella* sp. and *Escherichia coli*, but no infection was observed. Thus, a more diverse panel of bacteria could reveal a broader host range potential of the isolated phages.

*Klebsiella* CPS is known to be one of the main virulence factors. Its highly diverse chemical organization allows the bacterium to escape immune responses and antibiotics (25, 26). In addition, the severity of *K. pneumoniae* infections has been correlated with certain K-types (27, 28). It expresses on its surface two polysaccharides including LPS and capsule polysaccharide, which define the O-antigen group and the K-antigen type, respectively (25). Both structures can act as a receptor for *Klebsiella* phages. For many described *Klebsiella* phages, the depolymerase domain was characterized as a common primary RBP on CPS or exopolysaccharides (12, 18, 29). In this scenario, after degradation of the CPS by the depolymerase, the phage binds irreversibly to its secondary receptor before injecting its genetic material into the host (30). Consequently, some modifications of the capsule may alter the success of the infection. In fact, the emergence of bacterial resistance to phage induced by a mutation in genes involved in the generation of the CPS has been described (31, 32). Interestingly, no depolymerase domains were identified in the genome of the isolated phages, which seems to be a common feature in *Sugarlandvirus* (20). Furthermore, the number of identified RBPs is not of the same order as the number of susceptible K-types, unlike the previously described broad host range phages, whose mechanism of infection is based on CPS degradation (18). However, to completely eliminate this possibility, additional comprehensive analysis should be performed to determine the absence of depolymerase activity. Nevertheless, in most cases, the isolated phages vB_Kpn_K7PH164C4 and vB_Kpn_K30λ2.2 required CPS for successful infection. The remaining lytic activity of the phages observed in those cases could be explained by the co-existence of cap+ and cap− bacteria. In addition, we identified two genes that showed evidence of association with phage susceptibility. Previous research suggested that these genes may modulate the CPS phenotype, indicating that although CPS may not be the primary receptor, it remains a crucial structure in the context of phage infection. Interestingly, 13 of the 55 bacteria susceptible to any of the isolated phages did not possess either of the two identified genes. This observation implies the existence of additional infectivity factors not captured by our data. Our findings align with the potential role of the carbohydrate-binding module (CBM) domain identified in RBP β during the infection process. Previous research has reported proteins with hydrolytic activity against polysaccharides possessing this domain at their N-terminal end. This domain has been characterized as the binding site for extracellular polysaccharides, which, in this context, could be the LPS or CPS (33, 34). Thus, the mode of infection of the discovered *Sugarlandvirus* appears to be mainly dissociated from conventional depolymerase domains. Interestingly, these viruses display a CBM, which could potentially interact with a primary carbohydrate-based receptor. The elongated configuration of the protein could allow the phage to navigate around the capsule without needing to degrade it, giving it access to the secondary receptor. Further research is needed to elucidate the precise infection mechanism employed by the phages.

To characterize the host range of the phage, both liquid infection and spot test measurements were performed. We observed discrepancies in the results for phages vB_Kpn_K7PH164C4 and vB_Kpn_K30λ2.2 between the two methods. Phages with characteristics similar to those found here have been recently described (35). The RBPs identified in the broad host range S8/S9 phages presented a CBM instead of a depolymerase domain. However, a lower depolymerase activity was demonstrated for one of these phages. A similar phenomenon could explain some of the results obtained here, where a positive spot test was observed without lytic activity in liquid media. Moreover, the observed results could be due to lysis from without. In contrast, the cases where lytic activity was observed in liquid media but not in semi-solid media seemed less clear. This observation could be attributed to differential gene expression within the K-locus between the two media, resulting in recognition of the CPS in liquid media but not in semi-solid media (36). Despite sharing very similar genomic characteristics, phenotypic differences were observed between *Klebsiella* phages vB_Kpn_K7PH164C4 and vB_Kpn_K30λ2.2 on one side and vB vB_Kpl_K32PH164C1 on the other. The former phages could infect twice as many reference strains as the latter. The host recognition has been described as the most critical step in the phage infection process. Consequently, it was hypothesized that differences in the genes involved in host recognition were the origin of the phenotypic differences. Interestingly, the sequence similarity between the RBPs of the isolated phages did not match the observed phenotypic differences. Indeed, the RBPs showed a higher degree of similarity between vB_Kpn_K30λ2.2 and vB_Kpl_K32PH164C1 than between vB_Kpn_K30λ2.2 and vB_Kpn_K7PH164C4.

The isolated phages shared interesting features for phage therapy. The phages were predicted to be lytic, with no evidence of related lysogenic genes, and more importantly, the phages presented a broad host range. However, *Klebsiella* is known to emerge with resistance to phages within a few hours (19, 37). An interesting approach to reduce the emergence of resistant bacteria is to combine phages in a cocktail. There are multiple ways in which phage cocktails can tackle the problem of resistance emergence. Although the efficacy of phage cocktails comprising phages targeting the same receptor is questionable, as cross-resistance would easily arise after mutation of a single host gene, the use of phages targeting different receptors may delay or prevent the emergence of resistance (38). In this study, no delay in the emergence of resistance was observed when vB_Kpn_K7PH164C4 and vB_Kpn_K30λ2.2 phages were used in a cocktail. In addition, the accumulation of phage resistance mutations often has a fitness cost that can potentially reduce bacterial virulence (39). Moreover, direct or indirect interactions between phages may occur, which could enhance or hamper infectivity (19, 40). Cases have also been described in which the emergence of resistance to one phage can lead to susceptibility to another phage (41). Therefore, a good understanding of the RBP of the phage, its receptor, and potential interactions with other phages is esential for the successful design of a phage therapy. As mentioned, the emergence of resistance in *Klebsiella* after a few hours of phage incubation is a critical aspect in phage therapy since it represents one of the main limitations. In fact, neither vB_Kpn_K7PH164C4 nor vB_Kpn_K30λ2.2 was able to completely prevent the growth of the *Klebsiella* spp. tested for more than 6 h.

### Conclusions

In conclusion, we have presented new broad-range *Klebsiella* phages that show the broadest capsular-type host range to our knowledge. In this study, we have deciphered an undescribed host recognition process that relies on the expression of the capsule without its degradation in a classical manner. Understanding the infectivity process of phages is key to optimize their use in the context of phage therapy, so more efforts should be invested in unraveling the activity of *Sugarlandvirus*. The results obtained here reveal that these phages share interesting features that support their use in the clinic. However, due to the lack of regulation of the use of phages in biomedicine in some countries, further research in this field is still needed, with the hope of being able to make use of phages in the near future as an alternative to combat MDR bacteria.

## MATERIALS AND METHODS

### Bacterial strains

The bacteria used in this study include the 77 K-type reference strains of *Klebsiella* spp. obtained from the collection of the Statens Serum Institute (Copenhagen, Denmark) (Table S1). *K. pneumoniae* Aerogenes 4140 (K-type 7), *K. pneumoniae* 7824 (K-type 30), and *K. planticola* 6837 (K-type 32) were specifically used for phage isolation.

### Isolation, purification, and amplification of phages

Wastewater samples from the metropolitan area of Valencia (Spain) were used for phage hunting. Fifty milliliters of sewage water was taken and kept at 4°C until processing. The supernatant was centrifuged for 10 min at 4,000 rpm to remove bacteria and large particles and was filtered through 0.22-µm filters. Next, 1 mL of the filtered environmental sample was mixed with 200 µL of stationary *Klebsiella* LB cultures and 3.5 mL of top agar. The mixture was poured onto a plate with semi-solid LB and allowed to incubate overnight at 37°C for visualization of lytic plaques. Phage isolation was performed by three plaque-to-plaque passages, and the resulting phages were stored at −80°C. Phage amplification was performed in LB liquid cultures using the same bacterial strain from which they were isolated. For this purpose, exponential phase cultures of the targeted bacteria were inoculated with the isolated phage and grown at 37°C at 600 rpm for 3 h. After incubation, cultures were centrifuged twice at 13,000 rpm for 5 min to remove bacteria, and the resulting supernatant was collected and filtered through 0.22-μL filters. To obtain a high-titer culture, a centrifugation was performed at 30,000 × *g* for 2 h. The supernatant was discarded, and the pellet was resuspended in 200 µL of SM buffer.

### Transmission electron microscopy of the isolated phages

To obtain transmission electron micrographs of the isolated *Klebsiella* phages, a drop of each high-titer phage was deposited on a carbon-coated Formvar layer held by a copper grid. The sample was allowed to dry for 30 min, and excess liquid was removed. Finally, the phages were negatively stained with 2% phosphotungstic acid and examined under a transmission electron microscope.

### Assessment of phage infectivity

The three new *Klebsiella* phages were tested against the 77 *Klebsiella* reference strains by spot tests. For this purpose, a drop of 1 µL containing each phage at 10^7^ PFU was poured into a specific spot in semi-solid cultures of the target bacterium and incubated for 24 h at 37°C. Two replicates of all spots were performed, and in cases of incongruence, a third replicate was performed. A positive infection in the spot test corresponded to the formation of a clear plaque. Next, infections in liquid cultures were performed against the 77 *Klebsiella* reference strains for the phages vB_Kpn_K7PH164C4 and vB_Kpn_K30λ2.2. For the phage vB_Kpl_K32PH164C1, we executed the liquid assays for the *Klebsiella* reference strains in which the spot test revealed lytic activity in spots. For this purpose, 10^5^ PFU of each phage was inoculated to exponential growth phase bacterial cultures in 96-well plates at 37°C for 18 h in a plate reader (Multiskan). The optical density (OD_620_) was measured 100 times every 10.3 min to obtain the kinetics of the cultures (controls). In addition, non-inoculated cultures were also included as controls. Liquid infections were done in triplicate for all the reference strains.

### Combined phage treatment in liquid cultures

Phage cocktail treatment was assessed for the broad-range *Klebsiella* phages vB_Kpn_K7PH164C4 and vB_Kpn_K30λ2.2 in combined cultures. Infections (co-infections) in liquid cultures were performed using a cocktail encompassing both phages in the *Klebsiella* reference strains in which at least one phage was infective. For this purpose, 10^5^ PFU of each phage were inoculated to exponential growth phase bacterial cultures in 96-well plates at 37°C for 18 h in a plate reader (Multiskan), in parallel to the mono-infections.

### Evaluation of the bactericidal activity of phages

The bactericidal activity of broad-spectrum *Klebsiella* phages vB_Kpn_K7PH164C4 and vB_Kpn_K30λ2.2 was evaluated against susceptible bacterial strains in liquid media or semi-solid media. Phages were incubated in a volume of 500 µL at a concentration of 10⁶ PFU/mL with bacteria at an initial concentration of 10⁷ CFU/mL. After 4 h, 250 µL was used to measure the concentration after incubation. If the CFU count was not significantly lower than the control, the concentration was measured after 18 h in the remaining 250 µL.

### Generation of cap− bacteria

Spot tests were performed with phages from the in-house phage bank against the reference strains used in this study. Three phages presented a plaque during spot tests against reference strains K7, K11, and K64 surrounded by a halo, a hallmark of a depolymerase activity. Previous studies described loss of the capsule in resistant bacteria when incubated with a phage infecting through the capsule (42). Each of the reference strains K7, K11, and K64 was incubated with the corresponding halo-producing phage overnight. The precipitate was recovered after centrifugation and resuspended in 500 µL of LB medium. A 10-µL volume of the diluted sample was grown overnight in semi-solid medium to obtain cap− colonies. Candidate cap− colonies were picked and then grown in liquid medium until confluence. A volume of 500 µL was used to assess the absence of the capsule by microscopy. Briefly, the 500-µL volume was centrifuged 10 min at 14,000 rpm before removing the supernatant. The pellets were then resuspended in 20 µL of lysine and 210 µL of 2% formaldehyde solution. A 10-µL volume of the bacterial suspension was mixed in a drop of 10% negroin on a glass slide. After allowing the glass slide to dry, a 1% crystal violet solution was poured onto the slide and rinsed after 1 min. The final preparations were observed under the light microscope.

### Genomic analysis of the *Klebsiella* phages

High-titer phage lysates were used to extract DNA, followed by removal of non-encapsidated DNA using DNAse Turbo and capsid digestion by Proteinase K. DNA was then purified using the DNA Clean & Concentrator 5-Kit (Zymo) and sequenced with Illumina MiSeq technology, generating 250 paired-end reads. Kraken was used to analyze contaminants (43), while FastQC software (version 0.11.9, Babraham Bioinformatics) was used to assess sequencing quality. Genome assembly was performed *de novo* with SPAdes (version 3.13.0). The built-in read correction step of SPAdes was used for error correction and quality trimming before assembling Illumina reads (44). The quality of the generated assemblies was assessed using the QUAST tool (45). The similarities between the genomes were calculated with blastn with the parameters “-word_size 7 -reward 2 -penalty −3 -gapopen 5 -gapextend 2” (46). The whole assembled sequences were subjected to gene calling using Phanotate, Prodigal, Glimmer, and Genemarks. tRNA gene searching was performed with tRNAscan as implemented in the multiPhATE2 pipeline (47). By default, we allowed three start codons (codons start = {ATG, GTG, TTG}) and three termination codons (codons stop = {TAA, TAG, TGA}), and the default minimum length of an ORF was 90 nucleotides. For each predicted gene, functional annotation was done using blastn and blastp against different databases: National Center for Biotechnology Information (NCBI) viral genomes, NCBI virus protein database (August 2021), NCBI RefSeq protein database, Phage Annotation Tools and Methods (48) (available from http://www.phantome.org, last accessed 1 February 2022) (49), Prokaryotic Virus Orthologous Groups (50), Virus Orthologous Groups (VOGs), Swiss-prot protein sequence database (51), CAZy database (52), and the Millard database (Millard Lab database (available from http://millardlab.org, last accessed 1 March 2022) (53). Default cutoff values of blastn and blastp (60% identity) were used. Hidden Markov model profile searches were performed with hmmscan, phmmer, and jackmmer (54) against the pVOG and VOG hmm profile database. RBP sequences of the isolated phages were assessed using Uniref90 and small bfd databases to identify potential homologies to previous RBP α, RBP β, and RBP γ described. Depolymerase sequence was investigated using the Phyre2 web server (55).

To perform the taxonomic assignment of phages, two tools were used: PhaGCN, a semi-supervised learning model based on the construction of a knowledge graph by combining DNA sequences (56), and vConTACT (version 2.0), which applies a graph clustering algorithm to assign labels to unknown contigs (57). The genome plot comparing the *Klebsiella* phages was constructed with the R package gggenomes. The pairwise comparisons of all the nucleotide sequences were conducted using the Genome-BLAST Distance Phylogeny method (58) under settings recommended for prokaryotic viruses (57). The resulting intergenomic distances were used to infer a balanced minimum evolution tree with branch support via FASTME including SPR postprocessing (59). Branch support was inferred from 100 pseudo-bootstrap replicates each. Trees were rooted at the midpoint and visualized with FigTree (60). Taxon boundaries at the species, genus, and family levels were estimated with the OPTSIL program (61, 62), the recommended clustering thresholds, and an *F* value (fraction of links required for cluster fusion) of 0.5 (63). The life cycles of the phages were predicted using Natural Language Processing on the nucleotide sequences through the program PhageAI (64).

### Gene presence matrix of the K-locus in the reference strain

The K-locus sequence of the reference *Klebsiella* strains was downloaded from the NCBI database. A fraction of 64 of 77 of the sequences were available. Gene prediction and genome annotation were evaluated using the Prokka annotation tool (65). The GFF files generated by the program were used to calculate the gene presence-absence matrix with the Roary program (66).

### Receptor-binding protein analysis

The RBP sequences of the isolated phages were evaluated using the Uniref90 and small bfd databases to identify possible homologies to the α, β, and γ RBPs described above. The Rev-Trans1.4 codon aware aligner was used to generate the multiple nucleotide sequence alignment using the multiple protein sequence alignment constructed with Clustal Omega (version 1.2.4), along with the corresponding nucleotide sequences (67). In addition, multiple nucleotide sequence alignment (MSA) was analyzed to identify recombination events with RDP5 (68). The five methods implemented in the program with default settings were run to infer recombination events: RDP, GENECONV, MaxChi, BootScan, and SiScan.

Positive selection pressure was analyzed on each of the RBP sequences using different approaches as implemented by HYPHY (69). After removing the identified recombinants in the input MSAs, the following analyses were conducted. Gene-wide test for positive selection was assessed using BUSTED (70). The inference of non-synonymous and synonymous substitution rates on a per-site basis was conducted using a Bayesian approach with FUBAR (71) and a maximum-likelihood approach with FEL (72). Finally, mixed-effect maximum likelihood approach to test the hypothesis that individual sites have been subject to episodic positive or diversifying selection was evaluated with MEME (73).

### 3D protein structural modeling

3D models for RBPs were obtained with Alphafold version 2.1.0 collab notebook (74). The script was run with default parameters in relax mode. The predicted 3D structures were then visualized and annotated with ChimeraX (version 1.3) (https://www.rbvi.ucsf.edu/chimerax).

### Statistical analysis

The CFU count proceeded in triplicates. The differences in the numbers between conditions were evaluated using a one-tailed *t*-test. To evaluate differences of the liquid cultures when inoculated with the single phages or in a cocktail, we first calculated the AUC for every single replicate using Simpson’s rule. The variance between the set of AUCs was then compared through the *F*-test. Finally, the distribution of AUC under each conditions was tested through Student’s *t*-test. Lytic activity was evaluated by testing whether the AUC under a given condition was significantly smaller than the AUC of the control or if the AUC in the first 5 h was significantly smaller than the AUC of the control together with a positive signal at the spot test. Finally, the identification of genes in bacterial K-locus associated with phage susceptibility was assessed through an exact Fisher *t*-test. The *P* values were corrected using a false discovery rate with an alpha = 0.05. All the tests were conducted with scipy and sklearn packages in python.

## Data Availability

The sequences of the isolated phages are available in The European Nucleotide Archive under ENA PRJEB53659.

## SUPPLEMENTAL MATERIAL

Supplemental file 1 spectrum.04298-22-s0001.tif Fig. S1.

Supplemental file 2 spectrum.04298-22-s0002.tif Fig. S2

Supplemental file 3 spectrum.04298-22-s0003.tif Fig. S3

Supplemental file 4 spectrum.04298-22-s0004.tif Fig. S4

Supplemental file 5 spectrum.04298-22-s0005.docx Table S1

Supplemental file 6 spectrum.04298-22-s0006.docx Table S2

Supplemental file 7 spectrum.04298-22-s0007.docx Table S3

Supplemental file 8 spectrum.04298-22-s0008.docx Table S4

ASM does not own the copyrights to Supplemental Material that may be linked to, or accessed through, an article. The authors have granted ASM a non-exclusive, world-wide license to publish the Supplemental Material files. Please contact the corresponding author directly for reuse.
