# Supplementary figures and images for: Broad-range capsule-dependent lytic Sugarlandvirus against Klebsiella sp
Source: Microbiol Spectr. 2023 Oct 26;11(6):e04298-22. doi: 10.1128/spectrum.04298-22 (PMC10714931; doi:10.1128/spectrum.04298-22)

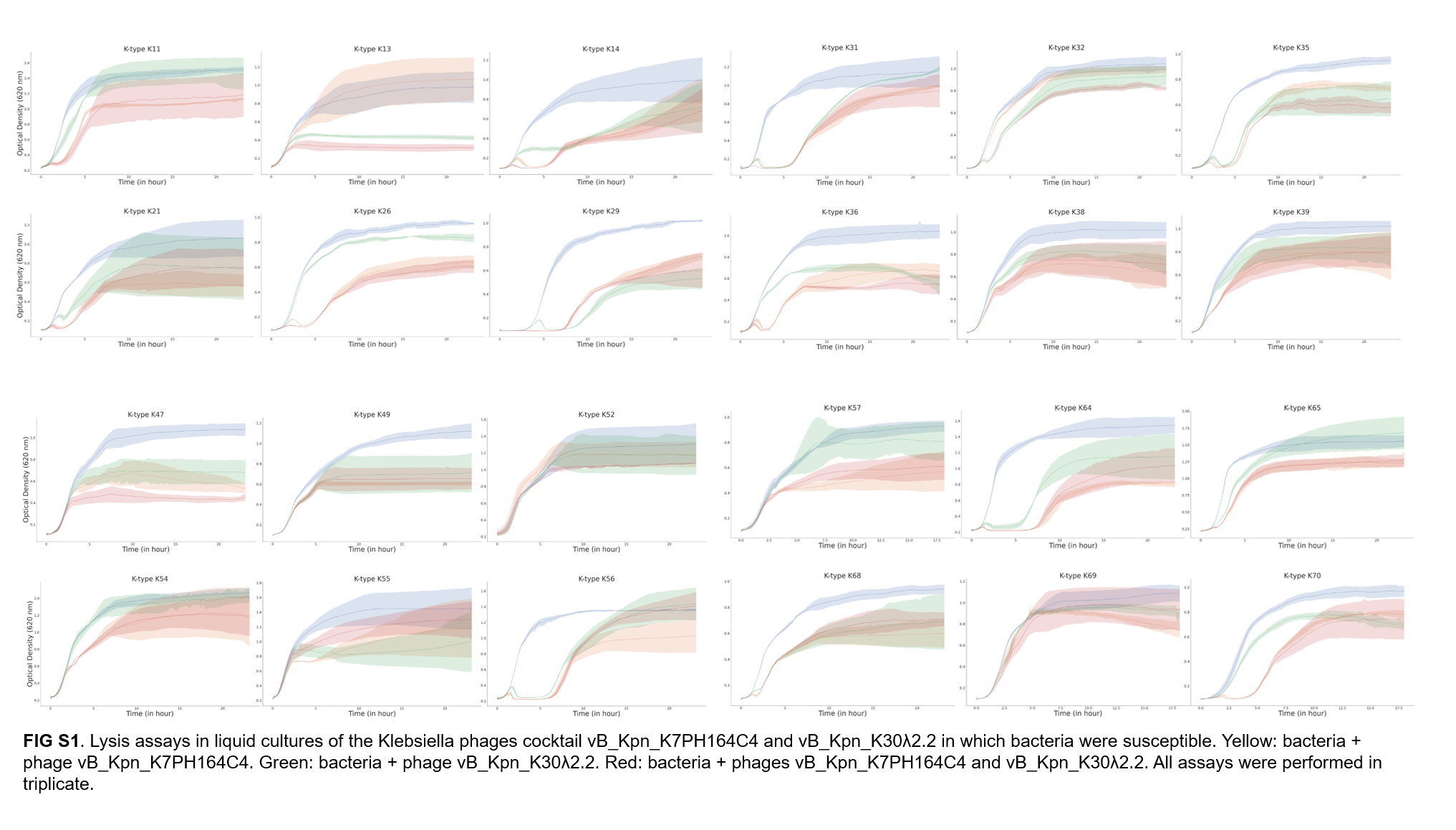

Supplement: Supplemental file 1 — Fig. S1. [file spectrum.04298-22-s0001.tif]

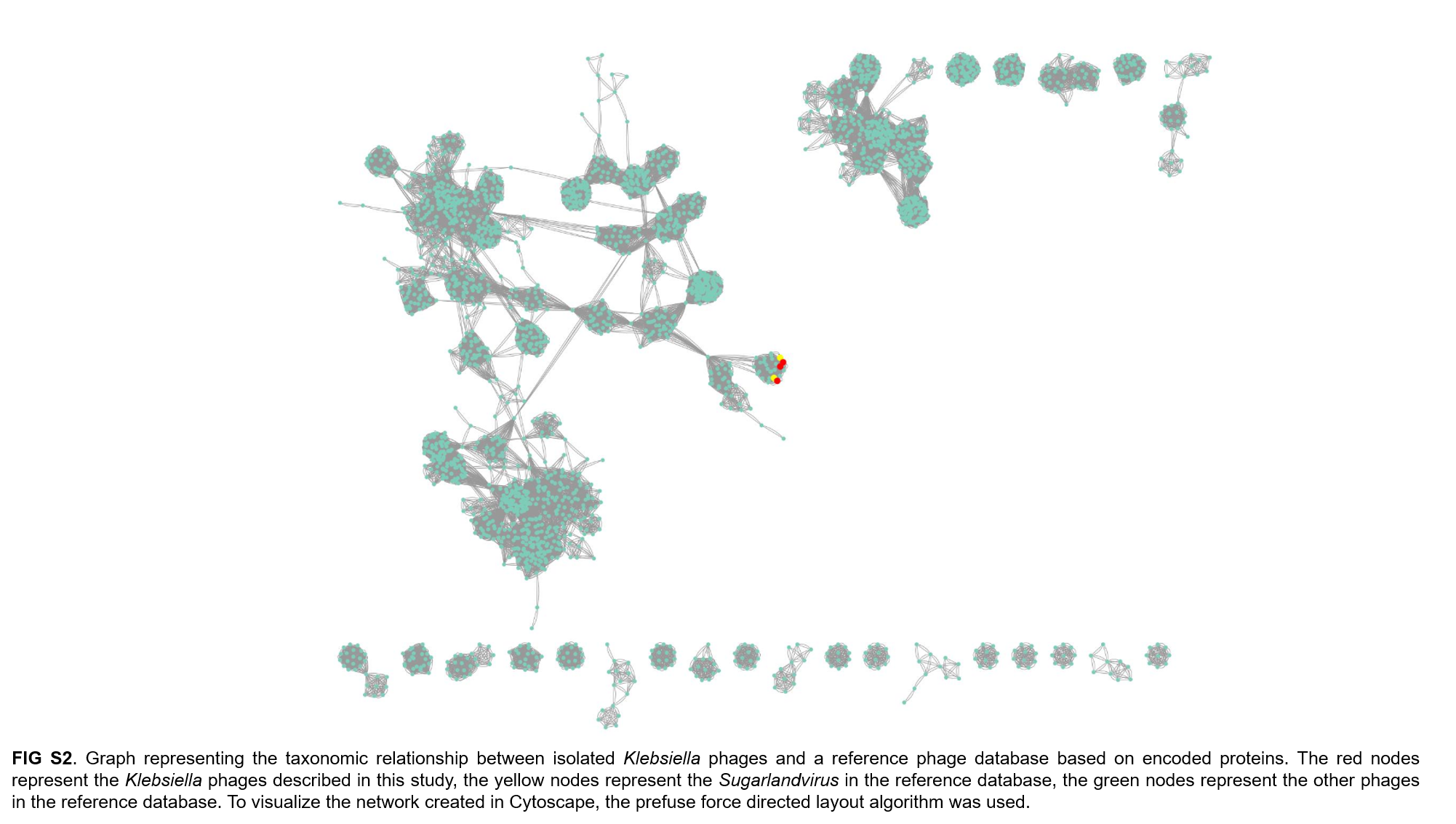

Supplement: Supplemental file 2 — Fig. S2 [file spectrum.04298-22-s0002.tif]

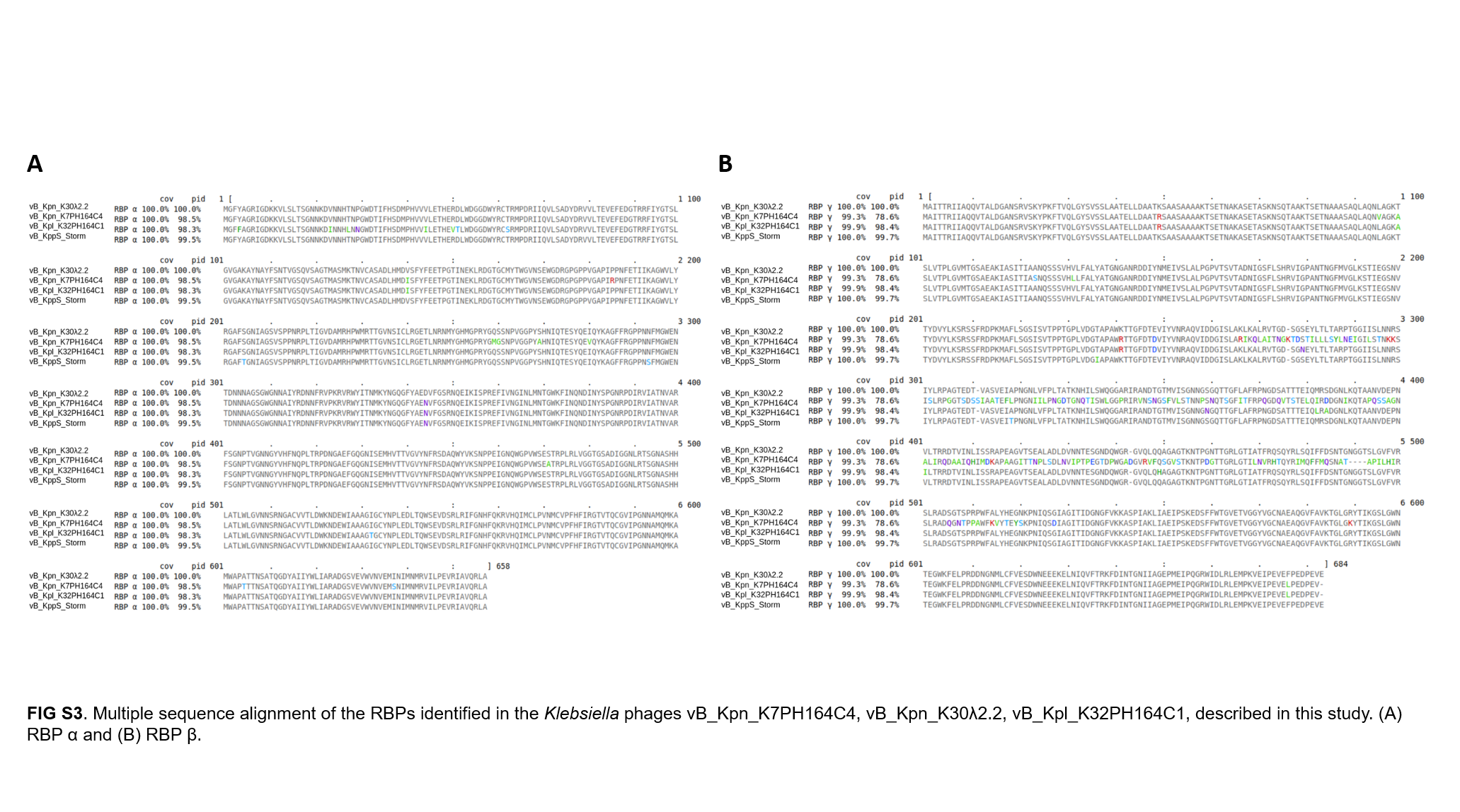

Supplement: Supplemental file 3 — Fig. S3 [file spectrum.04298-22-s0003.tif]

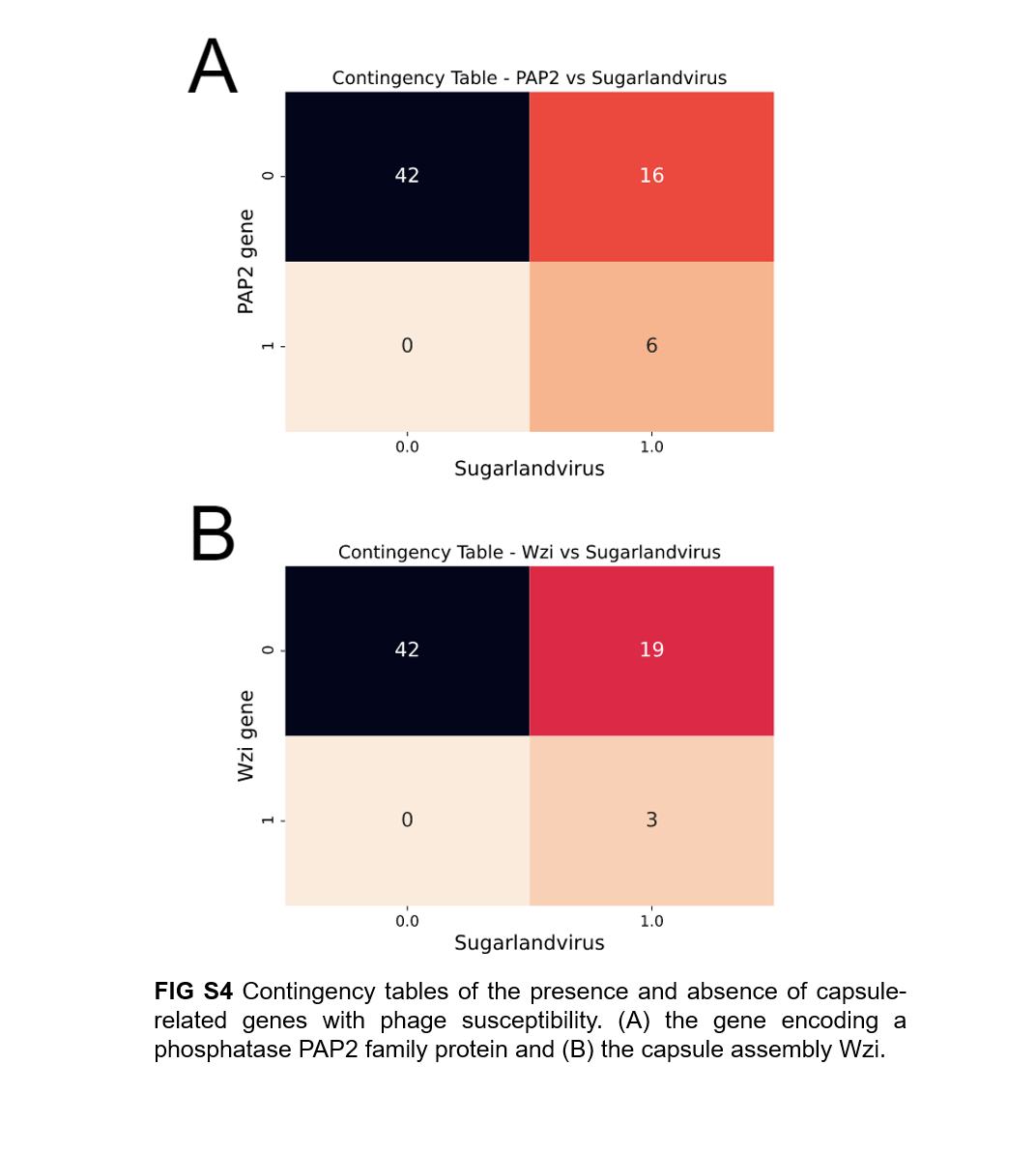

Supplement: Supplemental file 4 — Fig. S4 [file spectrum.04298-22-s0004.tif]
