# Supplementary material for: Broad-range capsule-dependent lytic Sugarlandvirus against Klebsiella sp
Source: Microbiol Spectr. 2023 Oct 26;11(6):e04298-22. doi: 10.1128/spectrum.04298-22 (PMC10714931; doi:10.1128/spectrum.04298-22)
Supplement: Supplemental file 5 — Table S1 [file spectrum.04298-22-s0005.docx]

**TABLE S1**. *Klebsiella* 77 K-type reference strains obtained from the Statens Serum Institute (Copenhagen, Denmark) collection used in this study.

| **K-type** | **Species** | **Strain number** |
| --- | --- | --- |
| **1** | *K. pneumoniae* | A 5054 |
| **2** | *K. pneumoniae* | B 5055 |
| **3** | *K. pneumoniae* | C 5046 |
| **4** | *K. ozaenae* | D 5050 |
| **5** | *K. ozaenae* | E 5051 |
| **6** | *K. pneumoniae* | F 5052 |
| **7** | *K. pneumoniae* | Aerogenes 4140 |
| **8** | *K. planticola* | Klebs. 1015 |
| **9** | *K. pneumoniae* | Klebs. 56 |
| **10** | *K. pneumoniae* | Klebs. 919 |
| **11** | *K. pneumoniae* | Klebs. 390 |
| **12** | *K. pneumoniae* | Klebs. 313 |
| **13** | *K. pneumoniae* | Klebs. 1470 |
| **14** | *K. planticola* | Klebs. 1193 |
| **15** | *K. pneumoniae* | Mich. 61 |
| **16** | *K. pneumoniae* | 2069/49 |
| **17** | *K. pneumoniae* | 2005/49 |
| **18** | *K. pneumoniae* | 1754/49 |
| **19** | *K. pneumoniae* | 293/50 |
| **20** | *K. pneumoniae* | 889/50 |
| **21** | *K. pneumoniae* | 1702/49 |
| **22** | *K. pneumoniae* | 1996/49 |
| **23** | *K. pneumoniae* | 2812/50 |
| **24** | *K. pneumoniae* | 1680/49 |
| **25** | *K. pneumoniae* | 2002/49 |
| **26** | *K. oxytoca* | 5884 |
| **27** | *K. pneumoniae* | 6613 |
| **28** | *K. pneumoniae* | 5758 |
| **29** | *K. oxytoca* | 5725y |
| **30** | *K. pneumoniae* | 7824 |
| **31** | *K. pneumoniae* | 6258 |
| **32** | *K. planticola* | 6837 |
| **33** | *K. pneumoniae* | 6168 |
| **34** | *K. pneumoniae* | 7522 |
| **35** | *K. planticola* | 7444 |
| **36** | *K. pneumoniae* | 8306 |
| **37** | *K. pneumoniae* | 8238 |
| **38** | *K. pneumoniae* | 8414 |
| **39** | *K. planticola* | 7749 |
| **40** | *K. pneumoniae* | 8588 |
| **41** | *K. oxytoca* | 6177 |
| **42** | *K. pneumoniae* | 1702 |
| **43** | *K. pneumoniae* | 2482 |
| **44** | *K. planticola* | 7730 |
| **45** | *K. pneumoniae* | 8464 |
| **46** | *K. pneumoniae* | 5281 |
| **47** | *K. pneumoniae* | 9682 |
| **48** | *K. planticola* | 1196 |
| **49** | *K. planticola* | 6115 |
| **50** | *K. pneumoniae* | 1303/50 |
| **51** | *K. pneumoniae* | 4715/50 |
| **52** | *K. pneumoniae* | 5759/50 |
| **53** | *K. planticola* | 1756/51 |
| **54** | *K. planticola* | Stanley |
| **55** | *K. pneumoniae* | 3985/51 |
| **56** | *K. planticola* | 3534/51 |
| **57** | *K. planticola* | 4425/51 |
| **58** | *K. planticola* | 636/52 |
| **59** | *K. planticola* | 2212/52 |
| **60** | *K. pneumoniae* | 4463/52 |
| **61** | *K. pneumoniae* | 5710/52 |
| **62** | *K. pneumoniae* | 5711/52 |
| **63** | *K. pneumoniae* | 5845/52 |
| **64** | *K. pneumoniae* | NCTC 8172 |
| **65** | *K. terrigena* | SW 4 |
| **66** | *K. oxytoca* | 438 (3a) |
| **67** | *K. pneumoniae* | 264 (1) |
| **68** | *K. pneumoniae* | 265 (1) |
| **69** | *K. pneumoniae* | 889 |
| **70** | *K. oxytoca* | 167 |
| **71** | *K. planticola* | 4349 |
| **72** | *K. planticola* | 1205 |
| **74** | *K. oxytoca* | 371 |
| **79** | *K. planticola* | 325 |
| **80** | *K. pneumoniae* | 708 |
| **81** | *K. pneumoniae* | 370 |
| **82** | *K. pneumoniae* | 3454-70 |
