# Supplementary material for: Broad-range capsule-dependent lytic Sugarlandvirus against Klebsiella sp
Source: Microbiol Spectr. 2023 Oct 26;11(6):e04298-22. doi: 10.1128/spectrum.04298-22 (PMC10714931; doi:10.1128/spectrum.04298-22)
Supplement: Supplemental file 6 — Table S2 [file spectrum.04298-22-s0006.docx]

**TABLE S2.** Colony forming units (CFU) count values after incubation of *Klebsiella* phages vB_Kpn_K7PH164C4 and vB_Kpn_K30λ2.2 in liquid media.

| ***Klebsiella* strain** | **Control (non infected)** | **SEM** | **K30λ2.2** | **SEM** | **K7PH164C4** | **SEM** | **cocktail** | **SEM** |
| --- | --- | --- | --- | --- | --- | --- | --- | --- |
| K7 | 1,77E+09 | 3,84E+08 | 7,33E+08 | 6,67E+07 | 1,36E+06 | 8,62E+05 |  |  |
| K11 | 4,63E+08 | 9,17E+07 | 3,97E+07 | 3,82E+07 | 9,67E+05 | 2,14E+05 |  |  |
| K13* | 3,53E+09 | 2,91E+08 | 9,57E+07 | 8,84E+06 | NA | NA | 1,56E+08 | 3,12E+07 |
| K14* | 3,43E+09 | 4,33E+08 | 1,60E+08 | 1,20E+08 | 1,56E+08 | 4,43E+07 |  |  |
| K21 | 1,03E+09 | 8,82E+07 | 5,23E+07 | 2,45E+07 | 1,30E+06 | 9,68E+04 |  |  |
| K26 | 1,20E+09 | 1,15E+08 | 3,11E+08 | 1,42E+08 | 1,42E+08 | 5,83E+07 |  |  |
| K29 | 3,40E+09 | 2,65E+08 | 6,33E+08 | 2,60E+08 | 2,19E+08 | 4,24E+07 |  |  |
| K30 | 9,33E+08 | 1,76E+08 | 2,35E+08 | 2,98E+07 | 1,50E+07 | 1,15E+06 |  |  |
| K31 | 9,39E+08 | 9,30E+08 | 1,00E+04 | 0,00E+00 | 1,73E+06 | 1,16E+06 |  |  |
| K32 | 2,23E+09 | 2,96E+08 | 6,00E+08 | 1,15E+08 | NA | NA |  |  |
| K35 | 9,33E+08 | 2,67E+08 | 1,77E+07 | 4,41E+06 | 3,00E+07 | 3,21E+06 |  |  |
| K36 | 7,00E+08 | 2,08E+08 | 2,77E+07 | 1,03E+07 | 6,52E+08 | 7,90E+07 |  |  |
| K38 | 6,33E+08 | 3,33E+07 | 7,00E+05 | 9,07E+04 | 8,00E+05 | 6,93E+04 |  |  |
| K39 | 6,53E+07 | 9,84E+06 | NA | NA | 6,77E+05 | 2,79E+05 |  |  |
| K47 | 1,55E+08 | 2,94E+07 | 1,33E+04 | 3,33E+03 | 1,33E+04 | 3,33E+03 | 1,00E+04 | 0,00E+00 |
| K49 | 1,47E+09 | 1,20E+08 | 9,67E+08 | 6,67E+07 | 1,27E+09 | 2,33E+08 |  |  |
| K52 | 1,23E+05 | 5,67E+04 | 6,70E+05 | 3,30E+05 | NA | NA |  |  |
| K54* | 3,20E+09 | 5,20E+08 | NA | NA | 1,13E+09 | 2,85E+08 |  |  |
| K55 | 1,54E+09 | 8,33E+07 | 1,72E+08 | 6,40E+07 | 1,31E+09 | 8,08E+07 |  |  |
| K56 | 1,08E+09 | 2,91E+07 | 1,00E+04 | 0,00E+00 | 1,37E+09 | 1,53E+07 |  |  |
| K57* | 2,63E+09 | 6,89E+08 | NA | NA | 4,67E+08 | 1,86E+08 |  |  |
| K64 | 1,93E+09 | 6,23E+07 | 1,00E+04 | 0,00E+00 | 2,40E+09 | 2,28E+08 |  |  |
| K65 | 2,13E+07 | 1,76E+06 | 1,97E+07 | 6,49E+06 | 8,44E+08 | 8,28E+08 |  |  |
| K68 | 5,33E+08 | 1,47E+08 | 3,03E+09 | 4,18E+08 | 1,33E+09 | 3,00E+07 |  |  |
| K69 | 1,08E+09 | 5,93E+07 | NA | NA | 1,52E+09 | 5,51E+07 |  |  |
| K70 | 8,60E+08 | 8,33E+07 | 1,00E+09 | 7,00E+07 | 1,64E+09 | 3,79E+07 |  |  |
|  |  |  |  |  |  |  |  |  |
| * 18 hours post infection |  |  |  |  |  |  |  |  |
| NA: non available |  |  |  |  |  |  |  |  |
