# Supplementary material for: Broad-range capsule-dependent lytic Sugarlandvirus against Klebsiella sp
Source: Microbiol Spectr. 2023 Oct 26;11(6):e04298-22. doi: 10.1128/spectrum.04298-22 (PMC10714931; doi:10.1128/spectrum.04298-22)
Supplement: Supplemental file 7 — Table S3 [file spectrum.04298-22-s0007.docx]

**TABLE S3**. Predicted ORFs in the genome of *Klebsiella* phages vB_Kpn_K7PH164C4, vB_Kpn_K30λ2.2, vB_Kpl_K32PH164C1, isolated in this study.

**Opening reading frames (ORFs) of phage vB_Kpn_K7PH164C4**

| ORFs | Start | Stop | Length (bp) | Orientation | Function |
| --- | --- | --- | --- | --- | --- |
| ORF 1 | 1 | 132 | 131 | + | Hypothetical Protein |
| ORF 2 | 181 | 708 | 527 | + | Hypothetical Protein |
| ORF 3 | 705 | 1052 | 347 | + | Hypothetical Protein |
| ORF 4 | 1905 | 2072 | 167 | + | Hypothetical Protein |
| ORF 5 | 2088 | 2276 | 188 | + | Hypothetical Protein |
| ORF 6 | 2444 | 2704 | 260 | + | Hypothetical Protein |
| ORF 7 | 3044 | 3448 | 404 | + | A2 Protein |
| ORF 8 | 3516 | 3785 | 269 | + | Hypothetical Protein |
| ORF 9 | 3782 | 4009 | 227 | + | Hypothetical Protein |
| ORF 10 | 4093 | 5769 | 1676 | + | A1 Protein |
| ORF 11 | 5834 | 6286 | 452 | + | Hypothetical Protein |
| ORF 12 | 6360 | 7076 | 716 | + | 5' Deoxynucleotidase |
| ORF 13 | 7233 | 7688 | 455 | - | Hypothetical Protein |
| ORF 14 | 7692 | 7916 | 224 | - | Hypothetical Protein |
| ORF 15 | 8030 | 10006 | 1976 | + | Tail Fiber Protein |
| ORF 16 | 10017 | 10490 | 473 | + | Terminase Small Subunit |
| ORF 17 | 10490 | 11806 | 1316 | + | Terminase Large Subunit |
| ORF 18 | 11935 | 12480 | 545 | + | Putative Endonuclease |
| ORF 19 | 12480 | 13691 | 1211 | + | Portal Protein |
| ORF 20 | 13688 | 14407 | 719 | + | Major Tail Protein |
| ORF 21 | 14604 | 15200 | 596 | + | Capsid And Scaffold Protein |
| ORF 22 | 15214 | 16596 | 1382 | + | Major Capsid Protein |
| ORF 23 | 16655 | 17167 | 512 | + | Tail Completion Protein |
| ORF 24 | 17167 | 17928 | 761 | + | Tail Completion Protein |
| ORF 25 | 17928 | 18413 | 485 | + | Tail Tube Terminator Protein |
| ORF 26 | 18437 | 19564 | 1127 | + | Major Tail Protein |
| ORF 27 | 19574 | 20461 | 887 | + | Tail Protein |
| ORF 28 | 20465 | 20887 | 422 | + | Hypothetical Protein |
| ORF 29 | 20859 | 21287 | 428 | + | Hypothetical Protein |
| ORF 30 | 21372 | 26450 | 5078 | + | Tail Length Tape-measure Protein |
| ORF 31 | 26565 | 27179 | 614 | + | Hypothetical Protein |
| ORF 32 | 27176 | 30022 | 2846 | + | Tail Length Tape-measure Protein |
| ORF 33 | 30023 | 40414 | 10391 | + | Tail Fiber Protein |
| ORF 34 | 40418 | 40837 | 419 | + | Baseplate Protein |
| ORF 35 | 40837 | 42876 | 2039 | + | Tail Fiber Protein |
| ORF 36 | 42921 | 43364 | 443 | - | Putative DeoxyUTP Pyrophosphatase |
| ORF 37 | 43364 | 44242 | 878 | - | Flap Endonuclease |
| ORF 38 | 44239 | 44721 | 482 | - | Putative Endonuclease |
| ORF 39 | 44721 | 46556 | 1835 | - | Putative Exonuclease |
| ORF 40 | 46540 | 47532 | 992 | - | Putative Exonuclease |
| ORF 41 | 47572 | 48345 | 773 | - | Hypothetical Protein |
| ORF 42 | 48348 | 48701 | 353 | - | Hypothetical Protein |
| ORF 43 | 48904 | 50262 | 1358 | - | Dna Helicase |
| ORF 44 | 50259 | 50846 | 587 | - | Hypothetical Protein |
| ORF 45 | 50749 | 53319 | 2570 | - | Dna Polymerase I |
| ORF 46 | 53387 | 54271 | 884 | - | Dna Primase |
| ORF 47 | 54268 | 55746 | 1478 | - | Dna Helicase |
| ORF 48 | 55831 | 56589 | 758 | - | D5 Protein |
| ORF 49 | 56592 | 57341 | 749 | - | Ndp-dependent Dna Ligase Subunit B |
| ORF 50 | 57541 | 58503 | 962 | - | Nad-dependent Dna Ligase Subunit A |
| ORF 51 | 58503 | 58766 | 263 | - | Hypothetical Protein |
| ORF 52 | 58702 | 58899 | 197 | - | Hypothetical Protein |
| ORF 53 | 58968 | 59276 | 308 | - | Ssdna-binding Transcriptional Regulator |
| ORF 54 | 59327 | 59623 | 296 | - | Hypothetical Protein |
| ORF 55 | 59636 | 60040 | 404 | - | D3 Protein |
| ORF 56 | 60119 | 60634 | 515 | - | Putative Hnh Endonuclease |
| ORF 57 | 60627 | 61322 | 695 | - | D2 Protein |
| ORF 58 | 61355 | 61639 | 284 | - | Hypothetical Protein |
| ORF 59 | 61626 | 64415 | 2789 | - | Dna Primase C |
| ORF 60 | 65014 | 65409 | 395 | - | Hypothetical Protein |
| ORF 61 | 65406 | 65807 | 401 | - | Hypothetical Protein |
| ORF 62 | 65794 | 65958 | 164 | - | Hypothetical Protein |
| ORF 63 | 65942 | 66775 | 833 | - | Sir2-like Protein |
| ORF 64 | 66775 | 66987 | 212 | - | Hypothetical Protein |
| ORF 65 | 67048 | 68877 | 1829 | - | Anaerobic Ribonucleoside-triphosphate Reductase |
| ORF 66 | 69187 | 69936 | 749 | + | Phoh-like Protein |
| ORF 67 | 69938 | 70051 | 113 | + | Hypothetical Protein |
| ORF 68 | 70066 | 70275 | 209 | + | Hypothetical Protein |
| ORF 69 | 70344 | 72728 | 2384 | + | Ribonucleotide Reductase A Subunit |
| ORF 70 | 72825 | 73985 | 1160 | + | Ribonucleotide Reductase B Subunit |
| ORF 71 | 73985 | 74548 | 563 | + | Dihydrofolate Reductase |
| ORF 72 | 74545 | 75399 | 854 | + | Putative Thymidylate Synthase |
| ORF 73 | 75406 | 75639 | 233 | + | Hypothetical Protein |
| ORF 74 | 75731 | 76012 | 281 | + | Hypothetical Protein |
| ORF 75 | 76012 | 76494 | 482 | + | Ribonuclease H |
| ORF 76 | 76494 | 76655 | 161 | + | Hypothetical Protein |
| ORF 77 | 76655 | 77005 | 350 | + | Hypothetical Protein |
| ORF 78 | 77059 | 77469 | 410 | + | Hypothetical Protein |
| ORF 79 | 77469 | 78473 | 1004 | + | Putative Metallopeptidase |
| ORF 80 | 78538 | 78999 | 461 | + | Putative Cell Wall Hydrolase Sleb |
| ORF 81 | 79559 | 79891 | 332 | + | Hypothetical Protein |
| ORF 82 | 80454 | 80780 | 326 | + | Hypothetical Protein |
| ORF 83 | 80783 | 80959 | 176 | + | Hypothetical Protein |
| ORF 84 | 81188 | 81481 | 293 | + | Hypothetical Protein |
| ORF 85 | 81615 | 81812 | 197 | + | Hypothetical Protein |
| ORF 86 | 81812 | 82021 | 209 | + | Hypothetical Protein |
| ORF 87 | 82032 | 82526 | 494 | + | Hypothetical Protein |
| ORF 88 | 84234 | 84428 | 194 | + | Hypothetical Protein |
| ORF 89 | 84415 | 84594 | 179 | + | Hypothetical Protein |
| ORF 90 | 85467 | 85739 | 272 | + | Hypothetical Protein |
| ORF 91 | 85760 | 86137 | 377 | + | Hypothetical Protein |
| ORF 92 | 86137 | 86352 | 215 | + | Hypothetical Protein |
| ORF 93 | 86424 | 86537 | 113 | + | Hypothetical Protein |
| ORF 94 | 87174 | 87380 | 206 | + | Hypothetical Protein |
| ORF 95 | 88144 | 88338 | 194 | + | Head To Tail Joining |
| ORF 96 | 88341 | 88679 | 338 | + | Hypothetical Protein |
| ORF 97 | 88679 | 88828 | 149 | + | Hypothetical Protein |
| ORF 98 | 89441 | 89557 | 116 | + | Hypothetical Protein |
| ORF 99 | 89676 | 89867 | 191 | + | Hypothetical Protein |
| ORF 100 | 89965 | 90321 | 356 | + | Hypothetical Protein |
| ORF 101 | 90312 | 90692 | 380 | + | Hypothetical Protein |
| ORF 102 | 90694 | 90870 | 176 | + | Hypothetical Protein |
| ORF 103 | 90965 | 91180 | 215 | + | Hypothetical Protein |
| ORF 104 | 91184 | 91456 | 272 | + | Hypothetical Protein |
| ORF 105 | 91456 | 91998 | 542 | + | Hypothetical Protein |
| ORF 106 | 91985 | 92212 | 227 | + | Hypothetical Protein |
| ORF 107 | 92199 | 92567 | 368 | + | Hypothetical Protein |
| ORF 108 | 92536 | 92802 | 266 | + | Hypothetical Protein |
| ORF 109 | 92765 | 93097 | 332 | + | Hypothetical Protein |
| ORF 110 | 93094 | 93303 | 209 | + | Hypothetical Protein |
| ORF 111 | 93305 | 93742 | 437 | + | Hypothetical Protein |
| ORF 112 | 93789 | 94112 | 323 | + | Hypothetical Protein |
| ORF 113 | 94105 | 94377 | 272 | + | Hypothetical Protein |
| ORF 114 | 94361 | 94804 | 443 | + | Hypothetical Protein |
| ORF 115 | 94924 | 95316 | 392 | + | Hypothetical Protein |
| ORF 116 | 95386 | 96138 | 752 | + | Portal Protein |
| ORF 117 | 96104 | 96565 | 461 | + | I Spanin |
| ORF 118 | 96804 | 97565 | 761 | + | Deoxynucleoside-5'-monophosphate Kinase |
| ORF 119 | 97575 | 98186 | 611 | + | Atp-dependent Clp Protease Proteolytic Subunit |
| ORF 120 | 98344 | 99006 | 662 | + | Putative Holin |
| ORF 121 | 99003 | 99416 | 413 | + | Putative Endolysin |
| ORF 122 | 99477 | 99893 | 416 | + | Hypothetical Protein |
| ORF 123 | 99966 | 100370 | 404 | + | Hypothetical Protein |
| ORF 124 | 100363 | 100647 | 284 | + | Putative Thioredoxin |
| ORF 125 | 100749 | 101153 | 404 | + | Hypothetical Protein |
| ORF 126 | 101153 | 101515 | 362 | + | Hypothetical Protein |
| ORF 127 | 101515 | 102345 | 830 | + | Putative Serine_threonine Protein Phosphatase |
| ORF 128 | 102356 | 102589 | 233 | + | Hypothetical Protein |
| ORF 129 | 102598 | 102966 | 368 | + | Hypothetical Protein |
| ORF 130 | 102969 | 103088 | 119 | + | Hypothetical Protein |
| ORF 131 | 103152 | 103580 | 428 | + | Hypothetical Protein |
| ORF 132 | 103662 | 103967 | 305 | + | Hypothetical Protein |
| ORF 133 | 103967 | 104248 | 281 | + | Hypothetical Protein |
| ORF 134 | 104245 | 104493 | 248 | + | Hypothetical Protein |
| ORF 135 | 104490 | 104831 | 341 | + | Hypothetical Protein |
| ORF 136 | 104824 | 105051 | 227 | + | Hypothetical Protein |
| ORF 137 | 105035 | 105502 | 467 | + | Hypothetical Protein |
| ORF 138 | 105462 | 105851 | 389 | + | Hypothetical Protein |
| ORF 139 | 105863 | 106150 | 287 | + | Hypothetical Protein |
| ORF 140 | 106200 | 106388 | 188 | + | Hypothetical Protein |
| ORF 141 | 106388 | 106864 | 476 | + | Hypothetical Protein |
| ORF 142 | 106842 | 107243 | 401 | + | Hypothetical Protein |
| ORF 143 | 107256 | 107765 | 509 | + | Hypothetical Protein |
| ORF 144 | 107778 | 108098 | 320 | + | Hypothetical Protein |
| ORF 145 | 108098 | 108862 | 764 | + | Hypothetical Protein |
| ORF 146 | 108864 | 109253 | 389 | + | Putative Endonuclease |
| ORF 147 | 110085 | 110234 | 149 | - | Hypothetical Protein |
| ORF 148 | 110983 | 111144 | 161 | - | Hypothetical Protein |
| ORF 149 | 111144 | 111284 | 140 | - | Hypothetical Protein |
| ORF 150 | 111357 | 111740 | 383 | + | Hypothetical Protein |
| ORF 151 | 111813 | 112778 | 965 | + | Hypothetical Protein |
| ORF 152 | 112844 | 113206 | 362 | + | Hypothetical Protein |

**Opening reading frames (ORFs) of phage vB_Kpn_K30λ2.2**

| ORFs | Start | Stop | Length (bp) | Orientation | Function |
| --- | --- | --- | --- | --- | --- |
| ORF 1 | 1 | 132 | 131 | + | Hypothetical Protein |
| ORF 2 | 174 | 713 | 539 | + | Hypothetical Protein |
| ORF 3 | 710 | 1057 | 347 | + | Hypothetical Protein |
| ORF 4 | 1911 | 2078 | 167 | + | Hypothetical Protein |
| ORF 5 | 2094 | 2282 | 188 | + | Hypothetical Protein |
| ORF 6 | 2450 | 2710 | 260 | + | Hypothetical Protein |
| ORF 7 | 3050 | 3454 | 404 | + | A2 Protein |
| ORF 8 | 3522 | 3791 | 269 | + | Hypothetical Protein |
| ORF 9 | 3788 | 4015 | 227 | + | Hypothetical Protein |
| ORF 10 | 4100 | 5776 | 1676 | + | A1 Protein |
| ORF 11 | 5822 | 6100 | 278 | + | Head Tail Joining |
| ORF 12 | 6129 | 6581 | 452 | + | Hypothetical Protein |
| ORF 13 | 6648 | 7370 | 722 | + | 5' Deoxynucleotidase |
| ORF 14 | 7516 | 7977 | 461 | - | Hypothetical Protein |
| ORF 15 | 7981 | 8205 | 224 | - | Hypothetical Protein |
| ORF 16 | 8319 | 10295 | 1976 | + | Tail Fiber Protein |
| ORF 17 | 10305 | 10778 | 473 | + | Terminase Small Subunit |
| ORF 18 | 10778 | 12094 | 1316 | + | Terminase Large Subunit |
| ORF 19 | 12223 | 12768 | 545 | + | Nicking Site-specific Endonuclease |
| ORF 20 | 12768 | 13979 | 1211 | + | Portal Protein |
| ORF 21 | 13976 | 14695 | 719 | + | Major Tail Protein |
| ORF 22 | 14895 | 15491 | 596 | + | Prohead Protease |
| ORF 23 | 15505 | 16887 | 1382 | + | Major Capsid Protein |
| ORF 24 | 16946 | 17458 | 512 | + | Tail Completion Protein |
| ORF 25 | 17458 | 18219 | 761 | + | Tail Completion Protein |
| ORF 26 | 18219 | 18704 | 485 | + | Tail Tube Terminator Protein |
| ORF 27 | 18728 | 19855 | 1127 | + | Major Tail Protein |
| ORF 28 | 19865 | 20752 | 887 | + | Minor Tail Protein |
| ORF 29 | 20756 | 21178 | 422 | + | Hypothetical Protein |
| ORF 30 | 21150 | 21578 | 428 | + | Hypothetical Protein |
| ORF 31 | 21663 | 26120 | 4457 | + | Tail Length Tape-measure Protein |
| ORF 32 | 26235 | 26849 | 614 | + | Distal Tail Protein |
| ORF 33 | 26846 | 29692 | 2846 | + | Tail Length Tape-measure Protein |
| ORF 34 | 29693 | 39304 | 9611 | + | Tail Fiber Protein |
| ORF 35 | 39308 | 39727 | 419 | + | Tail Protein |
| ORF 36 | 39727 | 41772 | 2045 | + | Tail Fiber Protein |
| ORF 37 | 41814 | 42257 | 443 | - | Deoxyuridine 5'-triphosphate Nucleotidohydrolase |
| ORF 38 | 42257 | 43135 | 878 | - | Ribonuclease H |
| ORF 39 | 43132 | 43614 | 482 | - | Endonuclease |
| ORF 40 | 43614 | 45449 | 1835 | - | Recombination-related Endonuclease |
| ORF 41 | 45433 | 46425 | 992 | - | Putative Recombination Endonuclease Subunit |
| ORF 42 | 46465 | 47238 | 773 | - | Exo1_bpt4 Exonuclease Subunit 1 |
| ORF 43 | 47241 | 47594 | 353 | - | Hypothetical Protein |
| ORF 44 | 47797 | 49155 | 1358 | - | Dna Helicase |
| ORF 45 | 49152 | 49739 | 587 | - | Hypothetical Protein |
| ORF 46 | 49642 | 52212 | 2570 | - | Dna Polymerase |
| ORF 47 | 52280 | 53164 | 884 | - | Hypothetical Protein |
| ORF 48 | 53161 | 54639 | 1478 | - | Dna Primase-helicase |
| ORF 49 | 54724 | 55482 | 758 | - | D5 Protein |
| ORF 50 | 55485 | 56234 | 749 | - | Ndp-dependent Dna Ligase Subunit B |
| ORF 51 | 56434 | 57396 | 962 | - | Nad-dependent Dna Ligase Subunit A |
| ORF 52 | 57396 | 57659 | 263 | - | Hypothetical Protein |
| ORF 53 | 57595 | 57792 | 197 | - | Hypothetical Protein |
| ORF 54 | 57861 | 58169 | 308 | - | Hypothetical Protein |
| ORF 55 | 58220 | 58516 | 296 | - | Hypothetical Protein |
| ORF 56 | 58529 | 58933 | 404 | - | D3 Protein |
| ORF 57 | 59008 | 59226 | 218 | - | Hypothetical Protein |
| ORF 58 | 59219 | 59914 | 695 | - | D2 Protein |
| ORF 59 | 59947 | 60231 | 284 | - | Hypothetical Protein |
| ORF 60 | 60218 | 63007 | 2789 | - | Obp-like Replication Origin Binding Protein And Helicase |
| ORF 61 | 63606 | 64001 | 395 | - | Hypothetical Protein |
| ORF 62 | 63998 | 64465 | 467 | - | Hypothetical Protein |
| ORF 63 | 64534 | 65436 | 902 | - | Sir2-like Protein |
| ORF 64 | 65369 | 65578 | 209 | - | Hypothetical Protein |
| ORF 65 | 65639 | 67468 | 1829 | - | Anaerobic Ribonucleoside-triphosphate Reductase |
| ORF 66 | 67778 | 68527 | 749 | + | Phoh-like Protein |
| ORF 67 | 68529 | 68642 | 113 | + | Hypothetical Protein |
| ORF 68 | 68657 | 68866 | 209 | + | Hypothetical Protein |
| ORF 69 | 68936 | 71320 | 2384 | + | Ribonucleotide Reductase A Subunit |
| ORF 70 | 71417 | 72577 | 1160 | + | Ribonucleotide Reductase B Subunit |
| ORF 71 | 72577 | 73140 | 563 | + | Dihydrofolate Reductase |
| ORF 72 | 73137 | 73991 | 854 | + | Putative Thymidylate Synthase |
| ORF 73 | 73998 | 74231 | 233 | + | Hypothetical Protein |
| ORF 74 | 74329 | 74604 | 275 | + | Hypothetical Protein |
| ORF 75 | 74604 | 75086 | 482 | + | Ribonuclease H |
| ORF 76 | 75086 | 75247 | 161 | + | Hypothetical Protein |
| ORF 77 | 75247 | 75597 | 350 | + | Hypothetical Protein |
| ORF 78 | 75651 | 76061 | 410 | + | Hypothetical Protein |
| ORF 79 | 76061 | 77065 | 1004 | + | Putative Metallopeptidase |
| ORF 80 | 77130 | 77591 | 461 | + | Putative Cell Wall Hydrolase Sleb |
| ORF 81 | 78905 | 79231 | 326 | + | Hypothetical Protein |
| ORF 82 | 79234 | 79410 | 176 | + | Hypothetical Protein |
| ORF 83 | 79639 | 79932 | 293 | + | Hypothetical Protein |
| ORF 84 | 80066 | 80263 | 197 | + | Hypothetical Protein |
| ORF 85 | 80251 | 80472 | 221 | + | Hypothetical Protein |
| ORF 86 | 80483 | 80977 | 494 | + | Hypothetical Protein |
| ORF 87 | 81603 | 81764 | 161 | + | Hypothetical Protein |
| ORF 88 | 82860 | 83051 | 191 | + | Hypothetical Protein |
| ORF 89 | 82939 | 83217 | 278 | + | Hypothetical Protein |
| ORF 90 | 83875 | 83991 | 116 | - | Hypothetical Protein |
| ORF 91 | 84126 | 84398 | 272 | + | Hypothetical Protein |
| ORF 92 | 84419 | 84796 | 377 | + | Hypothetical Protein |
| ORF 93 | 84796 | 85011 | 215 | + | Hypothetical Protein |
| ORF 94 | 85083 | 85196 | 113 | + | Hypothetical Protein |
| ORF 95 | 85499 | 85999 | 500 | + | Hypothetical Protein |
| ORF 96 | 87001 | 87195 | 194 | + | Head To Tail Joining |
| ORF 97 | 87198 | 87536 | 338 | + | Hypothetical Protein |
| ORF 98 | 87536 | 87685 | 149 | + | Hypothetical Protein |
| ORF 99 | 87909 | 88418 | 509 | + | Hypothetical Protein |
| ORF 100 | 88832 | 88933 | 101 | + | Hypothetical Protein |
| ORF 101 | 89052 | 89243 | 191 | + | Hypothetical Protein |
| ORF 102 | 89335 | 89697 | 362 | + | Hypothetical Protein |
| ORF 103 | 89688 | 90068 | 380 | + | Hypothetical Protein |
| ORF 104 | 90070 | 90237 | 167 | + | Hypothetical Protein |
| ORF 105 | 90230 | 90400 | 170 | + | Hypothetical Protein |
| ORF 106 | 90495 | 90710 | 215 | + | Hypothetical Protein |
| ORF 107 | 90714 | 90986 | 272 | + | Hypothetical Protein |
| ORF 108 | 90986 | 91528 | 542 | + | Hypothetical Protein |
| ORF 109 | 91515 | 91742 | 227 | + | Hypothetical Protein |
| ORF 110 | 91729 | 92097 | 368 | + | Hypothetical Protein |
| ORF 111 | 92066 | 92332 | 266 | + | Hypothetical Protein |
| ORF 112 | 92295 | 92627 | 332 | + | Hypothetical Protein |
| ORF 113 | 92624 | 92833 | 209 | + | Hypothetical Protein |
| ORF 114 | 92904 | 93227 | 323 | + | Hypothetical Protein |
| ORF 115 | 93220 | 93492 | 272 | + | Hypothetical Protein |
| ORF 116 | 93476 | 93919 | 443 | + | Hypothetical Protein |
| ORF 117 | 94039 | 94431 | 392 | + | Hypothetical Protein |
| ORF 118 | 94501 | 95253 | 752 | + | Hypothetical Protein |
| ORF 119 | 95219 | 95680 | 461 | + | I Spanin |
| ORF 120 | 95919 | 96680 | 761 | + | Deoxynucleoside-5'-monophosphate Kinase |
| ORF 121 | 96690 | 97301 | 611 | + | Atp-dependent Clp Protease Proteolytic Subunit |
| ORF 122 | 97459 | 98121 | 662 | + | Putative Holin |
| ORF 123 | 98118 | 98531 | 413 | + | Putative Endolysin |
| ORF 124 | 98592 | 99008 | 416 | + | Hypothetical Protein |
| ORF 125 | 99081 | 99485 | 404 | + | Hypothetical Protein |
| ORF 126 | 99478 | 99759 | 281 | + | Putative Thioredoxin |
| ORF 127 | 99864 | 100265 | 401 | + | Hypothetical Protein |
| ORF 128 | 100266 | 100628 | 362 | + | Hypothetical Protein |
| ORF 129 | 100628 | 101458 | 830 | + | Putative Serine_threonine Protein Phosphatase |
| ORF 130 | 101469 | 101702 | 233 | + | Hypothetical Protein |
| ORF 131 | 101711 | 102079 | 368 | + | Hypothetical Protein |
| ORF 132 | 102082 | 102201 | 119 | + | Hypothetical Protein |
| ORF 133 | 102265 | 102693 | 428 | + | Hypothetical Protein |
| ORF 134 | 102775 | 103080 | 305 | + | Hypothetical Protein |
| ORF 135 | 103080 | 103361 | 281 | + | Hypothetical Protein |
| ORF 136 | 103358 | 103606 | 248 | + | Hypothetical Protein |
| ORF 137 | 103603 | 103944 | 341 | + | Hypothetical Protein |
| ORF 138 | 103937 | 104164 | 227 | + | Hypothetical Protein |
| ORF 139 | 104148 | 104615 | 467 | + | Hypothetical Protein |
| ORF 140 | 104533 | 104964 | 431 | + | Hypothetical Protein |
| ORF 141 | 104976 | 105263 | 287 | + | Hypothetical Protein |
| ORF 142 | 105282 | 105785 | 503 | + | Hypothetical Protein |
| ORF 143 | 105763 | 106164 | 401 | + | Hypothetical Protein |
| ORF 144 | 106177 | 106686 | 509 | + | Hypothetical Protein |
| ORF 145 | 106699 | 107019 | 320 | + | Hypothetical Protein |
| ORF 146 | 107006 | 107941 | 935 | + | Hypothetical Protein |
| ORF 147 | 109684 | 109857 | 173 | - | Hypothetical Protein |
| ORF 148 | 109857 | 109997 | 140 | - | Hypothetical Protein |
| ORF 149 | 110070 | 110279 | 209 | + | Hypothetical Protein |
| ORF 150 | 110284 | 110442 | 158 | + | Hypothetical Protein |
| ORF 151 | 110515 | 111480 | 965 | + | Hypothetical Protein |
| ORF 152 | 111546 | 112043 | 497 | + | Hypothetical Protein |

**Opening reading frames (ORFs) of phage vB_Kpl_K32PH164C1**

| ORFs | Start | Stop | Length (bp) | Orientation | Function |
| --- | --- | --- | --- | --- | --- |
| ORF 1 | 1 | 132 | 131 | + | Hypothetical Protein |
| ORF 2 | 225 | 572 | 347 | + | Hypothetical Protein |
| ORF 3 | 572 | 811 | 239 | + | Hypothetical Protein |
| ORF 4 | 1443 | 1745 | 302 | + | Hypothetical Protein |
| ORF 5 | 1761 | 1949 | 188 | + | Hypothetical Protein |
| ORF 6 | 2115 | 2375 | 260 | + | Hypothetical Protein |
| ORF 7 | 2715 | 3122 | 407 | + | A2 Protein |
| ORF 8 | 3180 | 3407 | 227 | + | Hypothetical Protein |
| ORF 9 | 3490 | 5172 | 1682 | + | Phage A1 Protein Involved In Host Dna Degradation |
| ORF 10 | 5216 | 5494 | 278 | + | Head Tail Joining |
| ORF 11 | 5523 | 5975 | 452 | + | Hypothetical Protein |
| ORF 12 | 6042 | 6764 | 722 | + | Deoxynucleoside-5'-monophosphatase |
| ORF 13 | 6954 | 7400 | 446 | - | Hypothetical Protein |
| ORF 14 | 7404 | 7628 | 224 | - | Hypothetical Protein |
| ORF 15 | 7742 | 9718 | 1976 | + | Tail Fiber Protein |
| ORF 16 | 9711 | 10202 | 491 | + | Terminase Small Subunit |
| ORF 17 | 10202 | 11518 | 1316 | + | Terminase Large Subunit |
| ORF 18 | 11647 | 12192 | 545 | + | Nicking Site-specific Endonuclease |
| ORF 19 | 12192 | 13403 | 1211 | + | Portal Protein |
| ORF 20 | 13400 | 14119 | 719 | + | Major Tail Protein |
| ORF 21 | 14443 | 15039 | 596 | + | Prohead Protease |
| ORF 22 | 15053 | 16435 | 1382 | + | Major Capsid Protein |
| ORF 23 | 16494 | 17006 | 512 | + | Tail Completion Protein |
| ORF 24 | 17006 | 17767 | 761 | + | Tail Completion Protein |
| ORF 25 | 17767 | 18252 | 485 | + | Tail Tube Terminator Protein |
| ORF 26 | 18276 | 19403 | 1127 | + | Major Tail Protein |
| ORF 27 | 19413 | 20300 | 887 | + | Minor Tail Protein |
| ORF 28 | 20304 | 20726 | 422 | + | Hypothetical Protein |
| ORF 29 | 20698 | 21126 | 428 | + | Hypothetical Protein |
| ORF 30 | 21211 | 25668 | 4457 | + | Tape Measure Protein |
| ORF 31 | 25783 | 26397 | 614 | + | Distal Tail Protein |
| ORF 32 | 26394 | 29240 | 2846 | + | Tail Length Tape-measure Protein |
| ORF 33 | 29241 | 38861 | 9620 | + | Tail Fiber Protein |
| ORF 34 | 38865 | 39284 | 419 | + | Tail Protein |
| ORF 35 | 39284 | 41326 | 2042 | + | Tail Fiber Protein |
| ORF 36 | 41371 | 41814 | 443 | - | Deoxyuridine 5'-triphosphate Nucleotidohydrolase |
| ORF 37 | 41814 | 42692 | 878 | - | Ribonuclease H |
| ORF 38 | 42689 | 43171 | 482 | - | Endonuclease |
| ORF 39 | 43171 | 45006 | 1835 | - | Recombination-related Endonuclease |
| ORF 40 | 44990 | 45982 | 992 | - | Putative Recombination Endonuclease Subunit |
| ORF 41 | 46022 | 46795 | 773 | - | Exo1_bpt4 Exonuclease Subunit 1 |
| ORF 42 | 46798 | 47151 | 353 | - | Hypothetical Protein |
| ORF 43 | 47354 | 48712 | 1358 | - | Dna Helicase |
| ORF 44 | 48709 | 49296 | 587 | - | Hypothetical Protein |
| ORF 45 | 49199 | 51769 | 2570 | - | Dna Polymerase |
| ORF 46 | 51837 | 52721 | 884 | - | Dna Primase-helicase |
| ORF 47 | 52718 | 54196 | 1478 | - | Dna Primase-helicase |
| ORF 48 | 54281 | 55039 | 758 | - | D5 Protein |
| ORF 49 | 55042 | 55791 | 749 | - | Ndp-dependent Dna Ligase Subunit B |
| ORF 50 | 55991 | 57016 | 1025 | - | Nad-dependent Dna Ligase Subunit A |
| ORF 51 | 56953 | 57228 | 275 | - | Hypothetical Protein |
| ORF 52 | 57209 | 57346 | 137 | - | Hypothetical Protein |
| ORF 53 | 57430 | 57738 | 308 | - | Hypothetical Protein |
| ORF 54 | 57789 | 58085 | 296 | - | Hypothetical Protein |
| ORF 55 | 58098 | 58502 | 404 | - | D3 Protein |
| ORF 56 | 58577 | 58795 | 218 | - | Hypothetical Protein |
| ORF 57 | 58788 | 59483 | 695 | - | D2 Protein |
| ORF 58 | 59516 | 59800 | 284 | - | Hypothetical Protein |
| ORF 59 | 59787 | 62576 | 2789 | - | Obp-like Replication Origin Binding Protein |
| ORF 60 | 63175 | 63570 | 395 | - | Hypothetical Protein |
| ORF 61 | 63567 | 63968 | 401 | - | Hypothetical Protein |
| ORF 62 | 63955 | 64119 | 164 | - | Hypothetical Protein |
| ORF 63 | 64103 | 64936 | 833 | - | Sir2 Family Deacetylase |
| ORF 64 | 64936 | 65148 | 212 | - | Hypothetical Protein |
| ORF 65 | 65209 | 67038 | 1829 | - | Anaerobic Ribonucleoside-triphosphate Reductase |
| ORF 66 | 67348 | 68097 | 749 | + | Putative Phoh-like Protein |
| ORF 67 | 68099 | 68212 | 113 | + | Hypothetical Protein |
| ORF 68 | 68227 | 68436 | 209 | + | Hypothetical Protein |
| ORF 69 | 68505 | 70889 | 2384 | + | Aerobic Ribonucleoside Diphosphate Reductase |
| ORF 70 | 70986 | 72272 | 1286 | + | Ribonucleoside-diphosphate Reductase Subunit |
| ORF 71 | 72272 | 72835 | 563 | + | Dihydrofolate Reductase |
| ORF 72 | 72832 | 73686 | 854 | + | Thymidylate Synthase |
| ORF 73 | 73693 | 73926 | 233 | + | Hypothetical Protein |
| ORF 74 | 74018 | 74299 | 281 | + | Hypothetical Protein |
| ORF 75 | 74299 | 74781 | 482 | + | Putative Rnase H1 |
| ORF 76 | 74781 | 74942 | 161 | + | Hypothetical Protein |
| ORF 77 | 74942 | 75292 | 350 | + | Hypothetical Protein |
| ORF 78 | 75346 | 75756 | 410 | + | Hypothetical Protein |
| ORF 79 | 75756 | 76760 | 1004 | + | Putative Metallopeptidase |
| ORF 80 | 76825 | 77286 | 461 | + | Cell Wall Hydrolyse |
| ORF 81 | 77846 | 78166 | 320 | + | Hypothetical Protein |
| ORF 82 | 78606 | 78932 | 326 | + | Hypothetical Protein |
| ORF 83 | 78935 | 79111 | 176 | + | Hypothetical Protein |
| ORF 84 | 79340 | 79636 | 296 | + | Hypothetical Protein |
| ORF 85 | 79766 | 79963 | 197 | + | Hypothetical Protein |
| ORF 86 | 79963 | 80172 | 209 | + | Hypothetical Protein |
| ORF 87 | 80183 | 80677 | 494 | + | Hypothetical Protein |
| ORF 88 | 82250 | 82819 | 569 | + | Hnh Endonuclease |
| ORF 89 | 83675 | 83947 | 272 | + | Hypothetical Protein |
| ORF 90 | 83968 | 84345 | 377 | + | Hypothetical Protein |
| ORF 91 | 84345 | 84560 | 215 | + | Hypothetical Protein |
| ORF 92 | 84632 | 84745 | 113 | + | Hypothetical Protein |
| ORF 93 | 85048 | 85548 | 500 | + | Hypothetical Protein |
| ORF 94 | 85905 | 86105 | 200 | + | Hypothetical Protein |
| ORF 95 | 86737 | 86931 | 194 | + | Head Tail Joining |
| ORF 96 | 86934 | 87272 | 338 | + | Hypothetical Protein |
| ORF 97 | 87272 | 87421 | 149 | + | Hypothetical Protein |
| ORF 98 | 88243 | 88359 | 116 | + | Hypothetical Protein |
| ORF 99 | 88478 | 88669 | 191 | + | Hypothetical Protein |
| ORF 100 | 88767 | 89123 | 356 | + | Hypothetical Protein |
| ORF 101 | 89114 | 89494 | 380 | + | Hypothetical Protein |
| ORF 102 | 89484 | 89663 | 179 | + | Hypothetical Protein |
| ORF 103 | 89656 | 89826 | 170 | + | Hypothetical Protein |
| ORF 104 | 89921 | 90136 | 215 | + | Hypothetical Protein |
| ORF 105 | 90140 | 90412 | 272 | + | Hypothetical Protein |
| ORF 106 | 90412 | 90954 | 542 | + | Hypothetical Protein |
| ORF 107 | 90941 | 91168 | 227 | + | Hypothetical Protein |
| ORF 108 | 91155 | 91523 | 368 | + | Hypothetical Protein |
| ORF 109 | 91492 | 91758 | 266 | + | Hypothetical Protein |
| ORF 110 | 91721 | 92053 | 332 | + | Hypothetical Protein |
| ORF 111 | 92050 | 92259 | 209 | + | Hypothetical Protein |
| ORF 112 | 92261 | 92698 | 437 | + | Hypothetical Protein |
| ORF 113 | 92745 | 93068 | 323 | + | Hypothetical Protein |
| ORF 114 | 93061 | 93333 | 272 | + | Hypothetical Protein |
| ORF 115 | 93317 | 93760 | 443 | + | Hypothetical Protein |
| ORF 116 | 93880 | 94272 | 392 | + | Hypothetical Protein |
| ORF 117 | 94342 | 95094 | 752 | + | Hypothetical Protein |
| ORF 118 | 95060 | 95521 | 461 | + | I Spanin |
| ORF 119 | 95608 | 95763 | 155 | + | Hypothetical Protein |
| ORF 120 | 95760 | 96521 | 761 | + | Deoxynucleotide Monophosphate Kinase |
| ORF 121 | 96531 | 97142 | 611 | + | Atp-dependent Clp Protease Proteolytic Subunit |
| ORF 122 | 97300 | 97962 | 662 | + | Putative Holin |
| ORF 123 | 97959 | 98372 | 413 | + | Putative Endolysin |
| ORF 124 | 98433 | 98849 | 416 | + | Hypothetical Protein |
| ORF 125 | 98922 | 99326 | 404 | + | Hypothetical Protein |
| ORF 126 | 99319 | 99603 | 284 | + | Putative Thioredoxin |
| ORF 127 | 99705 | 100109 | 404 | + | Hypothetical Protein |
| ORF 128 | 100109 | 100471 | 362 | + | Hypothetical Protein |
| ORF 129 | 100471 | 101301 | 830 | + | Serine/threonine-protein Phosphatase 1 |
| ORF 130 | 101312 | 101545 | 233 | + | Hypothetical Protein |
| ORF 131 | 101554 | 101922 | 368 | + | Hypothetical Protein |
| ORF 132 | 101925 | 102044 | 119 | + | Hypothetical Protein |
| ORF 133 | 102108 | 102536 | 428 | + | Hypothetical Protein |
| ORF 134 | 102618 | 102923 | 305 | + | Hypothetical Protein |
| ORF 135 | 102923 | 103204 | 281 | + | Hypothetical Protein |
| ORF 136 | 103201 | 103449 | 248 | + | Hypothetical Protein |
| ORF 137 | 103446 | 103787 | 341 | + | Hypothetical Protein |
| ORF 138 | 103780 | 104007 | 227 | + | Hypothetical Protein |
| ORF 139 | 103991 | 104458 | 467 | + | Hypothetical Protein |
| ORF 140 | 104403 | 104807 | 404 | + | Hypothetical Protein |
| ORF 141 | 104818 | 105105 | 287 | + | Hypothetical Protein |
| ORF 142 | 105151 | 105627 | 476 | + | Hypothetical Protein |
| ORF 143 | 105605 | 106006 | 401 | + | Hypothetical Protein |
| ORF 144 | 106019 | 106528 | 509 | + | Hypothetical Protein |
| ORF 145 | 106541 | 106861 | 320 | + | Hypothetical Protein |
| ORF 146 | 106861 | 107166 | 305 | + | Hypothetical Protein |
| ORF 147 | 107163 | 107945 | 782 | + | Hypothetical Protein |
| ORF 148 | 108859 | 109323 | 464 | - | Hypothetical Protein |
| ORF 149 | 109646 | 109810 | 164 | - | Hypothetical Protein |
| ORF 150 | 109810 | 109923 | 113 | - | Hypothetical Protein |
| ORF 151 | 110131 | 110538 | 407 | + | Hypothetical Protein |
| ORF 152 | 110540 | 110740 | 200 | + | Hypothetical Protein |
