# Supplementary material for: Broad-range capsule-dependent lytic Sugarlandvirus against Klebsiella sp
Source: Microbiol Spectr. 2023 Oct 26;11(6):e04298-22. doi: 10.1128/spectrum.04298-22 (PMC10714931; doi:10.1128/spectrum.04298-22)
Supplement: Supplemental file 8 — Table S4 [file spectrum.04298-22-s0008.docx]

**TABLE S4**. Interproscan results of the RBP β in the *Klebsiella* phages vB_Kpn_K7PH164C4, vB_Kpn_K30λ2.2 and vB_Kpl_K32PH164C1.

| **Phage** | **Protein** | **Start** | **End** | **Hit** | **Interproscan** | **e-value** |
| --- | --- | --- | --- | --- | --- | --- |
| vB_Kpn_K7PH164C4 | RBP β | 1167 | 1315 | Galactose-bd-like_sf | IPR008979 | 1.38E-5 |
| vB_Kpn_K7PH164C4 | RBP β | 1917 | 1967 | Collagen | IPR008160 | 6.2E-7 |
| vB_Kpn_K30λ2.2 | RBP β | 1164 | 1313 | Galactose-bd-like_sf | IPR008979 | 5.95E-6 |
| vB_Kpn_K30λ2.2 | RBP β | 1911 | 1960 | Collagen | IPR008160 | 9.1E-7 |
| vB_Kpn_K30λ2.2 | RBP β | 3019 | 3077 | Collagen | IPR008160 | 1.2E-7 |
| vB_Kpl_K32PH164C1 | RBP β | 1164 | 1312 | Galactose-bd-like_sf | IPR008979 | 1.3E-5 |
| vB_Kpl_K32PH164C1 | RBP β | 1913 | 1968 | Collagen | IPR008160 | 4.2E-7 |
| vB_Kpl_K32PH164C1 | RBP β | 3022 | 3080 | Collagen | IPR008160 | 1.2E-7 |
